# Mouse models of patent ductus arteriosus (PDA) and their relevance for human PDA

**DOI:** 10.1002/dvdy.408

**Published:** 2021-08-14

**Authors:** Michael T. Yarboro, Srirupa H. Gopal, Rachel L. Su, Thomas M. Morgan, Jeff Reese

**Affiliations:** ^1^ Department of Cell and Developmental Biology Vanderbilt University Nashville Tennessee USA; ^2^ Department of Pediatrics Erlanger Health System Chattanooga Tennessee USA; ^3^ Division of Neonatology, Department of Pediatrics Vanderbilt University Medical Center Nashville Tennessee USA; ^4^ Division of Medical Genetics and Genomic Medicine, Department of Pediatrics Vanderbilt University Medical Center Nashville Tennessee USA

**Keywords:** congenital heart defect, ductus arteriosus, neonatal circulatory transition, vascular biology

## Abstract

The ductus arteriosus (DA) is a unique fetal vascular shunt, which allows blood to bypass the developing lungs in utero. After birth, changes in complex signaling pathways lead to constriction and permanent closure of the DA. The persistent patency of the DA (PDA) is a common disorder in preterm infants, yet the underlying causes of PDA are not fully defined. Although limits on the availability of human DA tissues prevent comprehensive studies on the mechanisms of DA function, mouse models have been developed that reveal critical pathways in DA regulation. Over 20 different transgenic models of PDA in mice have been described, with implications for human DA biology. Similarly, we enumerate 224 human single‐gene syndromes that are associated with PDA, including a small subset that consistently feature PDA as a prominent phenotype. Comparison and functional analyses of these genes provide insight into DA development and identify key regulatory pathways that may serve as potential therapeutic targets for the management of PDA.

## Key Findings

- Mouse models of patent ductus arteriosus (PDA) are informative for human PDA.
- Human single‐gene disorders with a PDA phenotype reveal interacting networks that contribute to regulation of DA patency and closure.
- Cross‐species correlations identify pathways of interest for development of PDA therapeutics.

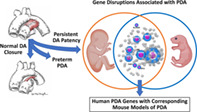

**Abbreviations**
AGTR1: angiotensin II receptor type 1
ALK1: activin like kinase 1
ASXL2: additional sex combs like 2
BMP: bone morphogenetic protein
BRG1: brahma‐related gene 1
BRM: brahma
CHD: congenital heart disease
CHF: congestive heart failure
COX‐1: cyclooxygenase 1
COX‐2: cyclooxygenase 2
DA: ductus arteriosus
DAVID: database for annotation visualization and integrated discovery
ECM: extracellular matrix
EP_4_
: prostanoid receptor EP_4_
GPC3: glypican 3
GPCR: G‐protein coupled receptor
HAND2: heart and neural crest derivatives‐expressed protein 2
HSPG: heparan sulfate proteoglycan
ILK: integrin‐linked kinase
ITGA2B: integrin alpha 2b
KO: knockout
LOX: lysyl oxidase
MF1: mesodermal/mesenchymal forkhead 1
MFH1: mesenchymal forkhead 1
MMP: matrix metalloproteinases
MTHFR: methylenetetrahydrofolate reductase
Myh11: myosin heavy chain 11
NC: neural crest
NF‐E2: nuclear factor erythroid 2
NT3: neurotrophin 3
OMIM: online Mendelian inheritance in man
PDA: patent ductus arteriosus
PDE3: phosphodiesterase 3
PGDH: 15‐hydroxyprostaglandin dehydrogenase
PGE_2_
: prostaglandin E_2_
PGT: prostaglandin transporter
PPI: protein‐protein interactions
Rbpj: recombinant signal binding protein for immunoglobulin kappa j region
RIM4: recombinant‐induced Mutation 4
SGBS: Simpson‐Golabi‐Behmel syndrome
SMC: smooth muscle cells
SNP: single nucleotide polymorphism
SRF: serum response factor
STRING: search tool for the retrieval of interacting genes/proteins
TAAD: thoracic aortic aneurysm and dissection
TFAP2B: transcription factor AP2 beta
TFGβ: transforming growth factor β
TNF: tumor necrosis factor
TRAF1: tumor necrosis factor receptor‐associated factor 1
TRKC: receptor tyrosine kinase
VNCC: vagal neural crest cell
VSMC: vascular smooth muscle cell
WT: wild‐type

## 1 INTRODUCTION

The ductus arteriosus (DA) is a fetal vessel, which shunts blood past the uninflated lungs, providing oxygenated blood from the placenta to the peripheral circulation and protecting the developing pulmonary vasculature in utero. At birth, increasing oxygen tension along with a decrease in prostaglandins and other vasodilatory mediators leads to constriction, closure, and subsequent fibromuscular transformation of the DA into the *ligamentum arteriosum*. The failure of the postnatal DA closure process may lead to the persistent patency of the ductus arteriosus (PDA), with potentially harmful consequences in newborns. PDA accounts for up to 10% of congenital heart disease (CHD) and is particularly problematic for preterm and especially low birthweight neonates.^1^, ^2^ In preterm infants born at 27 to 28 weeks gestation, 64% retain a patent DA at 7 days after birth, and among neonates born at 24 weeks, that figure increases to 87%.^3^ Options for management include pharmacological treatment with cyclooxygenase inhibitors, surgical ligation, interventional catheter‐based occlusion, or conservative management, each of which has potential for harm.^4^

Normal DA closure consists of a highly ordered series of biological steps involving different cell types, signaling pathways, and mechanical forces.^5^ Attempts to study these processes in preterm infants, while vital for advancing understanding and treatment of PDA, are limited by tissue availability and quality, as well as the nature of ex vivo and in vitro experiments. Large animal models have been used for centuries to study the anatomy, physiology, and pharmacology of the DA.^6^, ^7^ More recent studies on small animal models offer insights into DA embryology and function in more tractable laboratory species.^8^, ^9^ Rodent models of PDA have gained popularity due to their high fecundity, short gestation, and large litter sizes. The mouse is a robust and widely used mammalian model, which benefits from over a century of genetic methodology.^10^ The first transgenic models of PDA in mice were reported over 20 years ago.^11^, ^12^ Currently, there are 28 reported genetic mouse models of PDA, which provide insight into the role of specific ligands and receptors, structural or hematopoietic elements, and other molecular mediators of DA development and function. While some of these models may not be pertinent to the human DA, a comparison with human single‐gene syndromes associated with PDA may help identify relevant transcripts that warrant future analysis.

Human PDAs vary widely in their characteristics, severity, and underlying causes. A PDA in infancy may occur as part of a complex CHD or as an isolated anomaly. Isolated PDA occurs frequently in preterm infants, primarily as a result of developmental immaturity, which might not affect a given infant born at term. In contrast, a PDA in term infants is more likely to be associated with a genetic syndrome or a defined fetal embryopathy (eg, congenital rubella syndrome).^4^, ^13^ Both term and preterm PDAs may have a genetic component, with a 5% sibling recurrence rate^14^, ^15^ and a higher correlation between monozygotic twins compared with dizygotic twins.^16^, ^17^ While reports have varied, one twin study found that genetic factors and a common gestational environment contributed up to 76% of this variance. Studies on familial PDA and the offspring of consanguineous parentage provide genetic information on chromosome regions that confer risk for PDA.^18^, ^19^ In addition, candidate gene studies have identified genetic loci, which contribute to the syndromic forms of PDA such as *TFAP2B*, or whose sequence variants can contribute to isolated nonsyndromic cases of PDA.^20^, ^21^ Although the genetic predisposition for most PDAs is unknown, a robust understanding of the genes whose perturbation results in PDA may provide key insights into the development and function of the DA critical to the development of new and improved therapies.

In this review, we discuss the existing genetic mouse models of PDA and their potential implications for human DA biology. We probed multiple digital databases to identify single‐gene syndromes associated with PDA in humans. Gene ontology tools identified pathways and processes common between existing mouse models and human single‐gene syndromes (see Supporting Information).

## 2 MOUSE MODELS OF PDA

**FIGURE 1:**
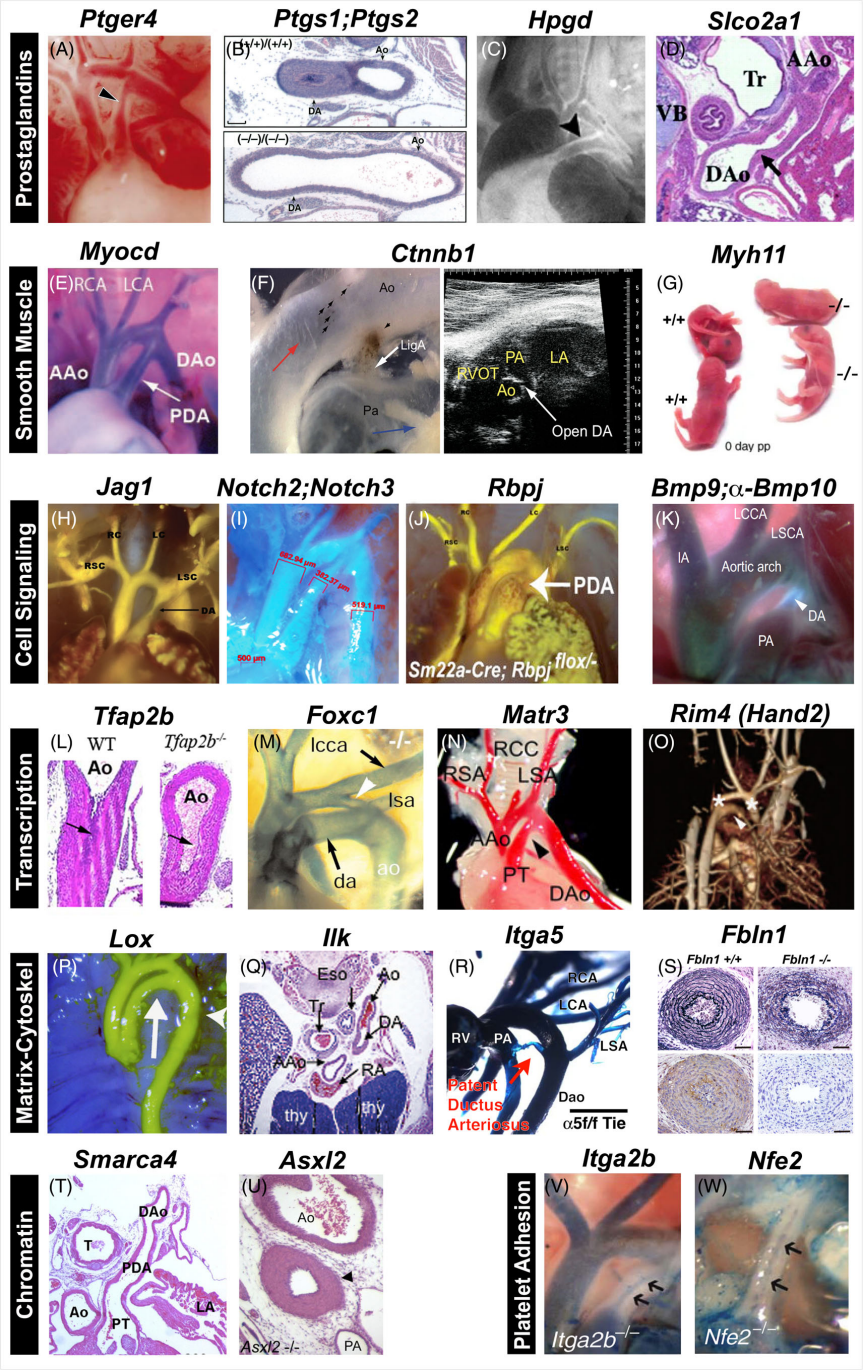
Representative images of various mouse knockout models exhibiting a patent ductus arteriosus (PDA) phenotype. PDA images (arrows, arrowheads) were obtained by whole‐mount or stained sections, as well as angiography and ultrasound. Images adapted or reproduced with permission. Citations from left‐to‐right, top‐to‐bottom (References 11, 22 [Copyright (2001) National Academy of Sciences],^23^, ^24^, ^25^, ^26^ (CC BY), and^27‐32^ (CC BY),^33^, ^34^, ^35^, ^36^, ^37^, ^38^, ^39^, ^40^, ^41^, ^42^)

Existing mouse models of PDA fall into several categories based on molecule type, localization, or pathway of action: components of the prostaglandin signaling pathway, proteins specific to smooth muscle cells (SMCs), proteins involved in developmental signaling, matrix and cytoskeletal components, platelet function, chromatin modifiers, and transcription factors. Representative images (Figure 1) and summary information (Table S1) for each model are provided.

### 2.1 Prostaglandin signaling

#### 2.1.1 Ptger4 *KO*

The prostaglandin E receptor EP_4_ is the canonical mediator of prostaglandin E2 (PGE_2_) effects in the DA. The EP_4_ receptor gene (*PTGER4*) is consistently enriched in both the mouse and human DA among various expression studies.^43^ The EP_4_ receptor is a G‐protein‐coupled receptor (GPCR), which is capable of signaling through both G‐α_s_ and G‐α_I_ G‐proteins giving it the ability to increase or decrease, respectively, the amount of cAMP in a cell, endowing potentially conflicting roles dependent on context.^44^ The EP_4_^−/−^ PDA phenotype was reported by three independent laboratories using distinct transgenic strategies.^11^, ^12^, ^45^ Nguyen et al. reported the first example of a mouse model of PDA in 1997. EP_4_^−/−^ mice had neonatal lethality accompanied by a widely patent DA and pulmonary edema (Figure 1A).^11^ This phenotype was observed by all three groups with varying penetrance. When the null allele was crossed into a mixed genetic background (B6D2 F1, C57BL/6, and DBA/2 cross), the uniformly lethal phenotype changed to 5% survival after one backcross and to 21% survival after four crosses.^11^ These data suggest that compensatory mechanisms exist for closing the DA and that genetic diversity may protect against genetic predisposition to PDA. The EP_4_^−/−^ PDA phenotype has been termed the “paradoxical PDA” due to its counterintuitive signaling, since the removal of a vasodilatory receptor is expected to foster constriction, not an inability to constrict.^46^ For this reason, it is suspected that EP_4_ may play an additional role in the DA, guiding vessel formation and remodeling. This has been further supported by findings that EP_4_ signaling is necessary for the production of hyaluronic acid in the DA, a process key for the movement of SMCs into the subendothelial space coinciding with permanent DA closure.^47^

#### 2.1.2 Ptgs1*;*Ptgs2 *double KO*

Cyclooxygenase‐1 (COX‐1) and cyclooxygenase‐2 (COX‐2) are the enzymes responsible for the production of PGE_2_, the primary ligand for the prostaglandin E (EP) receptors, including EP_4_.^44^ While COX‐1 and COX‐2 share similar functions, they often differ in localization and expression, and support different processes.^48^ COX‐1 is generally associated with tissue homeostasis and epithelial maintenance, whereas COX‐2 is typically associated with inflammation. The initial characterizations of mice with the targeted mutation of either the COX‐1 (*Ptgs1*) or COX‐2 (*Ptgs2*) genes did not reveal a DA phenotype. However, the generation of COX‐1^−/−^;COX‐2^−/−^ animals revealed a robust PDA phenotype and neonatal lethality.^49^ Animals showed the signs of congestive heart failure (CHF) similar to other PDA models. PDA was noted in both outbred CD‐1 mice^49^ and inbred C57Bl/6 mice (Figure 1B).^22^ While the link between prenatal exposure to COX inhibitors and PDA is established in both humans^50^, ^51^ and rodents,^52^, ^53^, ^54^ it remains paradoxical that the removal of a vasodilator results in dilation instead of constriction. In situ hybridization and PCR assays suggested that circulating PGE_2_ generated in peripheral tissues acts on PGE receptors in the DA via endocrine mechanisms.^49^ Moreover, pharmacologic studies suggest that prostaglandin ligand‐receptor signaling via the COX‐EP_4_ axis during specific gestational windows may play a novel role regulating DA development, in addition to their well‐known role in DA vasodilation.^53^

#### 2.1.3 Ptgs2 *KO*

Following the discovery of a PDA phenotype in COX‐1^−/−^;COX‐2^−/−^ mice, the COX‐1 and COX‐2 KO models were re‐examined. A PDA phenotype was found in COX‐2^−/−^ pups with 35% penetrance. Furthermore, while COX‐1^−/−^ offspring showed little or no PDA phenotype, the deletion of one COX‐1 allele increased the penetrance of the COX‐2^−/−^ PDA phenotype such that COX‐1^−/+^;COX‐2^−/−^ mice had 79% penetrance and COX‐1^−/−^;COX‐2^−/−^ mice displayed 100% penetrance.^55^ Additional studies utilized a targeted point mutation to generate mice expressing COX‐2 protein defective in COX function but preserving its peroxidase function (*Ptgs2* ^*Y385F*^). *Ptgs2* ^*Y385F*^ mice exhibited no PDA, which suggested the formation of COX‐1 and COX‐2^Y385F^ heterodimers.^56^ Despite the loss of COX‐2 functionality, *Ptgs2* ^*Y385F*^ mice produce enough PGE_2_ to maintain DA function, implying non‐catalytic COX‐2 heteromers are bound to functional COX‐1 partners. These findings suggest COX‐2 is the predominant COX isoform required for DA development and function, likely owing to its 10‐fold lower activating concentration,^57^, ^58^ and COX‐1 serves an auxiliary role, possibly through heterodimerization.

#### 2.1.4 Hpgd *KO*

Prostaglandins play key signaling roles in nearly all tissue types. In many contexts, prostaglandin‐mediated effects are regulated through catabolism by enzymes such as 15‐hydroxyprostaglandin dehydrogenase (PGDH). Mice hypomorphic for the PGDH gene (*Hpgd*) exhibit preterm labor associated with the genotype of both pup and dam. *Hpgd* ^*−/−*^ mice die neonatally with PDA (Figure 1C),^23^, ^59^ presumably related to the elevated levels of PGE_2_. In wild‐type (WT) mice, following the initiation of respiration, pulmonary vascular resistance falls as the DA constricts, redirecting blood through the newborn lungs, which express high levels of PGDH. PGDH catabolizes circulating PGE_2_, lowering serum levels leading to further DA closure. As expected, the postnatal administration of indomethacin can rescue *Hpgd* ^*−/−*^ animals by inhibiting prostaglandin synthesis and allowing PDA closure. Infants with mutations in the *HGPD* gene have multiple phenotypes, including PDA^60^ (Table S2).

#### 2.1.5 Slco2a1 *KO*

For PGDH to oxidize circulating prostaglandins, they must be internalized by the prostaglandin transporter (PGT) encoded by the *Slco2a1* gene. PGT is expressed with PGDH in the neonatal lung where it facilitates DA closure through reducing serum PGE_2_.^61^, ^62^ *Slco2a1* ^*−/−*^ mice are born in a Mendelian ratio but die shortly after birth with PDA and associated CHF (Figure 1D).^24^ *Slco2a1* hypomorphs survive a day longer, also dying with PDA. Histology of KO animals shows no differences in SMC composition or intimal thickening compared with WT. Similar to *Hpgd* ^*−/−*^ mice, both *Slco2a1* ^*−/−*^ and *Slco2a1* hypomorphs can be rescued with neonatal indomethacin. Mutations in the human *SLOC2A1* gene result in autosomal recessive hypertrophic osteoarthropathy and PDA in infants^24^, ^63^ (Table S2).

### 2.2 Smooth muscle cell specific

#### 2.2.1 Myocd *KO*

Myocardin is regarded as a master regulator of cardiac and SMC genes. Myocardin, encoded by the *Mycod* gene, is a transcriptional coactivator of the serum response factor (SRF) providing for the spatiotemporal expression of genes critical to cardiac and SMC cell fate.^64^, ^65^ Whereas global constitutive *Myocd* ^*−/−*^ mice die prior to E10.5, mice with neural crest (NC)‐selective deletion survive to term but die before P3 with PDA (Figure 1E).^25^ *Myocd* ^*−/−*^ DA tissue was deficient mature SMC markers such as *Acta2*, *Myh11*, and *Tagln*. These findings emphasize the importance of NC derivatives in DA formation and function.

#### 2.2.2 Ctnnb1 *KO*

Beta‐catenin is a cell‐cell adhesion protein and signal transducer for the Wnt pathway encoded by the *Ctnnb1* gene. Wnt signaling is key for many developmental processes, including the differentiation of vagal NC cells (VNCCs), which give rise to the SMCs of the DA.^66^, ^67^ Mice expressing constitutively activated beta‐catenin (*Ctnnb1Δex3*) were used to explore the VNCC role in establishing DA cell populations. These results confirmed that DA SMCs derive from three populations: the non‐pigmented non‐VNCC‐derived SMC1 (80%‐90%), the non‐pigmented VNCC‐derived population SMC2 (10%‐20%), and a very small number of pigmented VNCC‐derived melanoblasts (less than 1%).^26^ *Ctnnb1Δex3* mice exhibited shifts in cell population, with virtually all SMC2 cells replaced by melanoblasts and the SMC1 population unaffected. This shift was associated with PDA (Figure 1F). These findings suggest the Wnt‐driven phenotype of DA SMCs is key to proper formation and closure.

#### 2.2.3 Myh11 *KO*

The DA is a muscular artery with tone controlled by the constriction and relaxation of vascular SMCs (VSMCs). VSMC activity is driven by the contractile apparatus, comprised of specific actins, myosin heavy chains, and myosin light chains responsible for different phases of contraction. Smooth muscle myosin heavy chain 11 (*Myh11*) and other SMC genes are precociously expressed in DA SMCs compared to surrounding vessels.^68^, ^69^ *Myh11* ^−/−^ mice have delayed DA closure, taking 6 hours instead of 3 hours to close, and die as neonates unless their bladders are manually relieved (Figure 1G).^27^ Interestingly, PDA is not the cause of death although the left ventricle experiences hemodynamic overload similar to other models. While the DA was not assessed, isometric force measurements from the KO bladder tissue suggest muscle phenotypes, including delayed DA closure, may result from the loss of the transient high‐force phase 1 contraction in the KO. The sustained phase 2 contraction was unaffected and may explain the eventual DA closure.^27^ Infants with monoallelic mutations in *MYH11* can suffer both familial thoracic aortic aneurysm and PDA^70^ (Table S2), and MYH11 R712Q mutation causes diminished myosin motor elasticity.^71^

### 2.3 Developmental signaling

#### 2.3.1 Jag1 *SMC conditional KO*

JAG1 is a cell surface ligand, which binds Notch pathway receptors activating their downstream gene regulatory actions. Notch provides signaling between neighboring cells key for the proliferation, differentiation, and movement necessary for development and maintenance of the body.^28^ *Jag1* expression is normally limited to the endothelium but is found throughout the medial wall in mouse DAs. Interestingly, the endothelial‐specific deletion of *Jag1* resulted in embryonic death (~e10.5) with hemorrhages, vascular remodeling, and SMC differentiation defects.^28^ Subsequent studies of SMC conditional *Jag1* ^*−/−*^ mice revealed PDA and outflow tract defects (Figure 1H). Immunofluorescent staining revealed decreased the expression of mature SMC markers throughout the media of outflow tracts, with only SMCs contacting the endothelium appropriately differentiated. These findings suggest *Jag1*‐driven Notch signaling is key to the synthetic‐contractile fate of SMCs in the DA and outflow tracts. Furthermore, the SMC expression of *Jag1* seems key to the lateral transduction of differentiation signals from the endothelium. This signaling behavior is suggested to be unique to the DA and descending aorta. The PDA phenotype could be partially rescued with indomethacin within 12 hours after birth. Infants with *JAG1* mutations can suffer CHD, tetralogy of Fallot, and the more general Alagille syndrome, all associated with PDA^72^ (Table S2).

#### 2.3.2 Notch2 *KO/*Notch3 *Het*

Notch receptors (1‐4) detect surface ligands such as Jag1 on neighboring cells and drive nuclear localization. NOTCH2 and NOTCH3 are the predominant Notch receptors in the vasculature, NOTCH2 being more globally expressed. While the deletion of either receptor results in vascular defects, those associated with *Notch2* are considerably more severe.^29^ *Notch2* ^−/−^ mice have a partial phenotype, with ~40% dying postnatally with PDA (Figure 1I). Interestingly, *Notch2* ^*−/−*^;*Notch3* ^+/−^ mice all die with PDA. *Notch2* ^−/−^;*Notch3* ^+/−^ mice also have dilated aortic segments and decreased the medial expression of mature SMC markers. These data are consistent with the known role of Notch signaling in mature SMC differentiation. *Notch2* ^+/−^;*Notch3* ^−/−^ animals showed no PDA phenotype or neonatal death, indicating *Notch2* may be more critical for SMC differentiation in the DA. Infants with monoallelic NOTCH2 and NOTCH3 mutations may suffer from Hajdu‐Cheney syndrome^73^ and lateral meningocele syndrome,^74^ respectively, both associated with PDA (Table S2).

#### 2.3.3 Rbpj *SMC conditional KO*

The recombinant signal binding protein for the immunoglobulin kappa J region (*Rbpj*) is a key downstream transcriptional regulator of the Notch pathway. RBPJ acts as a repressor of gene expression but becomes an activator when bound to a Notch protein. Following their work on the *Jag1* ^−/−^ PDA, Gridley and colleagues created SMC‐specific conditional *Rbpj* ^*−/−*^ mice using the same *Tagln‐cre* driver as their previous model.^75^ As expected, the SMC‐specific *Rpbj* ^−/−^ mice die neonatally with PDA and decreased expression of mature SMC markers in the DA media (Figure 1J).^30^ Interestingly, whereas *Jag1* ^−/−^ mice could be rescued with neonatal indomethacin, only one of nine *Rbpj* ^−/−^ animals were rescued. This stronger phenotype indicates there may be other Notch ligands that contribute to the eventual activation of RPBJ and mature SMC differentiation.

#### 2.3.4 Gdf2 *KO* anti‐Bmp10

Bone morphogenetic proteins (BMPs) are members of the transforming growth factor beta (TGFβ) superfamily and play key roles in guiding tissue architecture throughout the body. BMP9 and BMP10 have both been shown to bind the activin receptor‐like kinase 1 (ALK1) on the endothelium of blood vessels, suggesting a role in vascular disease.^31^ *Bmp10* ^*−/−*^ (BMP10) mice die with cardiac defects in mid‐gestation, while *Gdf2* ^*−/−*^ (BMP9) mice are viable.^31^ Interestingly, while the *Gdf2* ^−/−^ DA is occluded enough to prevent flow at P5, its lumen is not completely filled with intimal cells, such as the WT, and red blood cells can be observed. This phenotype is exacerbated by the administration of a neutralizing anti‐BMP10 antibody on P1 and P3. *Gdf2* ^*−/−*^ anti‐BMP10‐treated mice achieve temporary DA constriction on P0 and P3, indistinguishable from WT, but show a partially patent lumen at P5 lined with endothelial cells, red blood cells, and an island of intimal cells (Figure 1K). These findings were not observed with the anti‐BMP10 treatment of WT animals, or at later time points (P3, P5), suggesting a narrow window when BMP function is critical for the fibromuscular transformation of the DA into the *ligamentum arteriosum*. Recombinant BMP9 and BMP10 were found to increase the expression of *Ptgs2* and *Has2* mRNA, which encodes hyaluronic acid synthase (HAS), where HA is a key component for matrix deposition and cell movement. Furthermore, at P3, *Gdf2* ^−/−^ anti‐BMP10‐treated mice lacked the matrix deposition key to DA fibrosis and *ligamentum arteriosum* formation. Thus, *Gdf2* ^−/−^ anti‐BMP10‐treated mice are one of the few mouse models with abnormalities in the second, anatomical closure phase of permanent DA remodeling.

#### 2.3.5 Gpc3 *KO*

Glypican‐3 (*Gpc3*) is a heparan sulfate proteoglycan (HSPG), which plays a key role in cardiac development. Glypicans attach themselves to cell surfaces through glycophosphatidylinositol linkages, where they bind and modify various ligands, modulating cell signaling. Previous studies suggest that *Gpc3* specifically may interact with BMP, Hedgehog, Wnt, and FGF signaling pathways^76^ and is widely expressed throughout vertebrate development. However, human loss‐of‐function *GPC3* mutations result in a rare congenital overgrowth syndrome associated with CHD; Simpson‐Golabi‐Behmel syndrome (SGBS). Similarly, when *Gpc3* ^*−/−*^ mice were examined, they were found to have multiple defects, including PDA. While *Gpc3* ^−/−^ mutants exhibited a delay in coronary vascular plexus formation and subsequent reduction in sonic hedgehog mRNA expression consistent with GPC3 acting as a co‐receptor for FGF9, it is unclear whether these signaling disruptions could contribute to a PDA phenotype or even whether the PDA observation in this model is biologically significant. *Gpc3*'s association with both BMP and Wnt signaling family members and the presence of a PDA phenotype in infants suffering SGBS,^77^ provide plausibility that *Gpc3* plays a role in DA function (Table S2).

### 2.4 Transcription

#### 2.4.1 Tfap2b *KO*

The importance of the transcription factor AP2 beta (*TFAP2b*) in DA function was first observed in human clinical populations. Mutations in *TFAP2b* lead to Char syndrome, a NC disorder associated with craniofacial abnormalities and PDA.^20^, ^32^, ^78^ Similarly, single nucleotide polymorphisms/mutations in *TFAP2b* are associated with nonsyndromic PDA.^79^, ^80^ Subsequently, a *Tfap2b* ^*−/−*^ mouse model revealed kidney disorders, delayed closure of the DA, and neonatal death (Figure 1L).^81^ These findings were corroborated by a recent CRISPR *Tfap2b* KO. In situ hybridization revealed that *Tfap2b* specifically labels DA SMCs with tight borders until E18.5.^82^ Interestingly, the vessel wall of *Tfap2b* ^−/−^ DAs showed no significant changes in morphology or elastin deposition, but in situ hybridization revealed a significant decrease in calponin, a robust marker of mature SMCs at E18.5.^83^ KO animals were also found to have decreased expression of *Hif2a* and *Et‐1*, suggesting a *Tfap2b*‐driven signaling cascade, which plays a key role in DA oxygen‐sensing mechanisms at birth^83^ (Table S2).

#### 2.4.2 Foxc1 *KO*

Mesenchymal forkhead 1 (MFH1 or FOXC2) and mesodermal/mesenchymal forkhead 1 (MF1 or FOXC1) are both forkhead family transcription factors, which share a nearly identical DNA binding domain as well as overlapping embryonic expression in the paraxial mesoderm, mesenchyme, and endothelium of the branchial arches. MFH1 and MF1 KO mice die prenatally and perinatally with a spectrum of cardiovascular and skeletal defects.^33^, ^84^ Interestingly, when *Mfh1* ^*tm1+/−*^ and *Mf1* ^*lacZ+/−*^ mice are crossed to obtain *Mfh1* ^*tm1+/−*^;*Mf1* ^*lacZ+/−*^ double heterozygotes, nonallelic noncomplementation leads to a similar spectrum of cardiovascular defects including PDA (Figure 1M)^33^ accompanied by prenatal and perinatal death. While PDA was not detected in *Mfh1* ^−/−^ mice, it was detected in *Mf1* ^−/−^ mice. Sectioning of *Mf1* ^−/−^ mice at d10.5 revealed fully formed and symmetrical aortic arches, indicating that *MF1* expression is not required for aortic arch formation. *Mf1* and *Mfh1* are thought to mediate signaling between the endothelium of the intima and the NC‐derived mesenchyme of the media, likely related to cell fate determinations. It makes sense that *MF1* expression decreases in the DA following closure, as both of these populations die out. Infants with mutations in *FOXC1* may suffer Axenfeld‐Rieger syndrome,^85^ which is associated with PDA (Table S2).

#### 2.4.3 Matr3 *KO*

Matrin3 is a nuclear matrix protein that is associated with distal myopathy 2, including vocal cord and pharyngeal muscle weakness. Genetic examination of a novel proband exhibiting developmental delay and cardiovascular defects including PDA revealed mutations in both *AHDC1* and Matrin 3 (*MATR3*). While the *AHDC1* mutation is likely the source of developmental delay, creation of a genetrap construct in exon 13 of the mouse *Matr3* gene revealed a key role for *Matr3* in cardiovascular development.^34^ Homozygous *Matr3* ^Gt‐ex13^ mice show early embryonic death (most by 4.5dpc, all by 8.5dpc). *Matr3* ^*Gt‐ex13*^ heterozygotes showed a spectrum of cardiovascular defects similar to the human proband including PDA in 12% of heterozygotes (Figure 1N). Immunohistochemistry showed the localization of *Matr3* in both the SMCs and endothelial cells of the large arteries. These data, considered with Matr3's proposed role in stabilizing select mRNAs, indicate a key role in the proper development of the outflow tracts. Infants with mutations in *MATR3* suffer from various phenotypes, including PDA^34^ (Table S2).

#### 2.4.4 Hand2 *trisomy;* Rim4 *mouse*

The human disorder 4q+ is a syndrome resulting from a triplicated region of the human chromosome 4. 4q+ results in varied phenotypes including delays in growth and cognition, physical deformities, and CHD including PDA. Interestingly, a mouse model with an analogous trisomy mutation, the recombinant‐induced mutation 4 (Rim4) mouse was discovered allowing studies into which genes might be responsible. Rim4 heterozygous mice and 4q+ humans are both trisomic for the heart and NC derivatives‐expressed protein 2 (*Hand2*) gene, which codes a member of the basic helix‐loop‐helix family of transcription factors associated with cardiovascular development and defects. Rim4 mice are generally unwell with 80% of those on a C57Bl/10J background dying neonatally. In addition, these mice were found to have PDA among other deformities (Figure 1O).^35^ Interestingly, these symptoms were ameliorated when Rim4 mice were crossed with a *Hand2* KO line to correct the genomic dosage of *Hand2*. *Hand2* was generally found to be necessary for proper formation of the ventricles and outflow tracts, consistent with its involvement with NC cells, although a mechanism of action is unknown.

### 2.5 Matrix/cytoskeleton

#### 2.5.1 Lox *KO*

Extracellular matrix (ECM) composition is critical for establishing both the mechanical properties and cell identity of blood vessels. Lysyl oxidase (*Lox*) encodes an enzyme responsible for the crosslinking of elastin and collagen, as well as influencing proliferation and cell fate. *Lox* ^−/−^ mice are born with abnormally formed outflow tracts, thoracic aortic aneurysm, and dissection (TAAD) and die as neonates with ruptured diaphragms, impaired airways, and PDA (22%) (Figure 1P).^36^ Closer examination of the ascending and descending aortas indicated disrupted elastin fiber formation and region‐specific changes in biomechanical properties. Regional changes in the expression of ECM, matrix metalloproteinases (MMP), and SMC cell cycle genes within the ascending and descending aorta suggest Lox‐mediated matrix crosslinking plays a critical role in DA development and function.

#### 2.5.2 Ilk *KO*

Integrin‐linked kinase (ILK) is a protein, which localizes to the integrins of the membrane‐associated dense plaques, where it uses its kinase domain to foster downstream signal transduction in response to force transduction signals between the contractile apparatus and ECM. ILK is critical for both polarization of the epiblast and vasculogenesis, resulting in embryonic lethality for *Ilk* ^−/−^ and endothelial‐specific *Ilk* ^*−/−*^ mice.^37^ To investigate ILK's role in vascular signal transduction, SMC‐specific *Ilk* ^−/−^ mice were created (*Sm22‐cre* ^*+*^;*Ilk* ^*Fl/Fl*^), which showed extremely dilated thoracic aortic aneurysms (up to 50% of the thorax) and PDA with associated perinatal lethality (Figure 1Q).^37^ Histological analysis revealed disruptions in the normal spindle‐like morphology and circumferential orientation of VSMCs and ablation of the elastin layer characteristic of elastic arteries. Morphogenic changes in outflow tract anatomy could be detected by e12.5. Notably, other NC‐associated defects were not observed. Immunohistochemical labeling for mature SMC‐specific markers indicated a loss in contractile SMC phenotype in the *Sm22‐cre* ^*+*^;*Ilk* ^*Fl/Fl*^ KO vessels. Together, these data suggest a critical role for *Ilk* in proper outflow tract development.

#### 2.5.3 Itgα5 *and* Itgαv *KOs*

Integrins are heterodimeric cell adhesion receptors, which mediate responses to ECM ligands. Integrins α5 and αv are the primary receptors for fibronectin and support angiogenesis by allowing endothelial cells to assess their environment. KOs of fibronectin and various integrins result in embryonic lethality, β1 integrin KOs being preimplantation lethal. Interestingly, only endothelial‐specific KO of *Itgα5* and *Itgαv* produced severe outflow tract defects. While only 4% of *Itgα5* ^−/−^;*Itgαv* ^−/−^ animals survived to adulthood, one adult displayed PDA. Subsequently, PDA was discovered in several *Itgα5* ^−/−^;*Itgαv* ^*+/−*^ animals of mixed genetic background (Figure 1R).^38^ Of the *Tie2‐cre* ^*+*^;*Itgα5* ^*flox/flox*^ WT mice examined at 10 to 20 weeks, 9/10 had PDA and half succumbed before weaning. Interestingly, these mice were on a C57BL/6 N7 background, whereas *Tie2‐cre* ^*+*^;*Itgα5* ^*flox/−*^ mice on a 129S4:C57BL/6 background, lacked PDA. This suggests strain specific modifiers modulate DA phenotypes. In addition, PDA afflicted adult mice of 10 to 20 weeks but may also contribute to premature loss of littermates. This suggests the loss of *Itgα5* may result in PDAs of varying severity, some hemodynamically tolerable. This discrepancy may result from background modifiers.

#### 2.5.4 Fbln1 *KO*

Fibulin‐1 (FBLN1) is a glycoprotein, which binds ECM proteins and participates in directed cell migration during development. Interestingly, *Fbln1* upregulation is reported in rat DA following EP_4_ stimulation. *Ptger4* ^*−/−*^ mice also have decreased *Fbln1* expression, suggesting EP_4_ stimulation may guide *Fbln1* expression. Furthermore, when *Fbln1* ^−/−^ mice were generated, 7/7 pups showed PDA 6 hours after birth with complete closure in controls (Figure 1S).^39^ *Fbln1* ^−/−^ mice also had decreased intimal thickening, where VSMCs migrate through the internal elastic lamina into the subendothelial space, facilitating DA closure. Thus, the disruption of VSMC migration in the *Fbln1* ^−/−^ DA and potentially the *Ptger4* ^−/−^ DA may contribute to PDA.

### 2.6 Chromatin

#### 2.6.1 Smarca4 *KO*

Brahma (BRM) and Brahma‐related gene 1 (BRG1; encoded by the *Smarca4* gene) are members of the SWI/SNF complex, an ATP‐dependent chromatin remodeling complex thought to play a role in SMC differentiation. While global *Smarca4* ^*−/−*^ mice die around implantation, SMC‐specific *Smarca4* ^*−/−*^ mice revealed ventricular septal defect and PDA (33% of offspring), resulting in CHF and neonatal death (Figure 1T).^40^ While *Smarca4* ^+/−^ offspring also show PDA (10%), possession of functional *Brm* alleles appears to be protective. Mature SMC gene expression was also lost in the GI tract and bladder. These data support independent roles for *Smarca4* and *Brm* in the differentiation of SMCs relevant for DA function.

#### 2.6.2 Asxl2 *KO*

The additional sex combs like 2 (*Asxl2*) gene encodes a putative polycomb group protein likely responsible for maintaining epigenetic gene repression through complex assembly. The exact mechanisms are debated.^86^ All three ASXL proteins (1, 2, and 3) are expressed in the outflow tracts, ASXL2 being the most enriched. *Asxl2* ^−/−^ mice in a C57BL/6 background die neonatally with PDA and severe cyanosis (98.2%), and other CHD (22%) (Figure 1U).^41^ Despite PDA, histology of WT and KO tissues was indistinguishable, suggesting *Asxl2*'s role in DA closure is nonstructural. Interestingly, *Asxl2* ^*−/−*^ mice on a mixed C57BL/6;129Sv genetic background lacked PDA and neonatal death, highlighting the strain‐dependence of PDA.

### 2.7 Platelet aggregation

#### 2.7.1 Itga2b *KO*

Platelet aggregation is thought to support DA occlusion due to the remodeling of endothelial and subendothelial SMCs during permanent DA closure. Disrupted endothelial surfaces provide access to collagen and therefore binding surfaces for activated platelets. The integrin alpha 2b (*Itga2b*) gene encodes a preprotein, which is processed to create subunits for the integrin alpha 2b/beta 3 receptor, which contributes to platelet aggregation. Interestingly, 31% of *Itga2b* ^−/−^ mice showed PDA 12 hours post‐delivery (Figure 1V).^42^ *Itga2b* ^−/−^ mice also exhibited a 26% reduction in luminal platelet accumulation neonatally. This decrease seems to disrupt either the thrombotic occlusion of the DA or platelet‐derived signaling involved in permanent closure.

#### 2.7.2 Nfe2 *KO*

The nuclear factor erythroid 2 (*Nfe2*) gene encodes an essential component of the NF‐E2 protein complex, which regulates megakaryocyte differentiation and, subsequently, platelet production. Similar to *Itga2b* ^−/−^, *Nfe2* ^−/−^ mice present with PDA 12 hours after delivery, though more frequently (70%), with 100% closure among WT littermates (Figure 1W).^42^ *Nfe2* ^−/−^ mice also had reduced platelet accumulation in the neonatal DA and decreased luminal proliferation. The *Nfe2* ^−/−^ PDA was unresponsive to indomethacin, further complicating prostaglandin's role in DA closure. Together, the *Itga2b* ^*−/−*^ and *Nfe2* ^−/−^ models suggest a role for platelet aggregation in murine DA closure. While the studies of platelets and DA closure in mice are limited, extensive clinical research has had mixed findings in humans. Several studies found associations between thrombocytopenia and PDA outcomes^42^, ^87^ or treatment failure.^88^ Others suggest that thrombocytopenia does not contribute to PDA^89^ and is not associated with an increased incidence of PDA,^90^, ^91^ and that transfusions of platelets have no effect on PDA closure.^92^

## 3 MOUSE MODELS OF PREMATURE DA CLOSURE

### 3.1 *Ntf3* KO

Neurotrophin 3 (*Nt3*) is a neuronal growth factor, which activates the receptor tyrosine kinase TRKC, supporting survival, and differentiation. Interestingly, *TrkC* is expressed in the non‐neuronal tissues of the heart and outflow tracts, as well as NC cells, suggesting *Nt3* may contribute to cardiovascular development. *Nt3* ^−/−^ animals show variable but severe CHD.^93^ Interestingly, all *Nt3* ^−/−^ animals show the premature closure of the DA in utero. While mechanisms are unknown, this is likely related to changes in survival or differentiation of the DA's NC‐derived population.

### 3.2 *Gja5* KO; *Gja1* Heterozygous

Gap junctions like connexins 40 (CX40/*Gja5*) and 43 (CX43/*Gja1*) contribute to cardiac conduction by facilitating electrical coupling through the movement of ions. While CX40 and CX43 serve similar functions, they vary in expression and are differentially dispensable for cardiac formation and survival, with *Gja1* ^−/−^ being nonviable.^94^, ^95^ Interestingly, crossing *Gja5* and *Gja1* KO lines indicates additive effects of connexin deficiency on cardiac conduction.^95^ The *Gja5* ^*−/−*^;*Gja1* ^*+/−*^ offspring are particularly interesting, as they are nonviable and show premature constriction of the DA at e18.5, in conjunction with severe CHD.

## 4 PHARMACOLOGICAL MODELS IN MICE

In addition to genetic models, pharmacological models that stimulate or inhibit particular pathways have proven valuable for interrogating PDA. An example is the use of the selective COX‐1 and COX‐2 inhibitors. Prolonged treatment of dams with COX‐1 and COX‐2 inhibitors during late gestation (D15‐D18) leads to PDA, whereas acute treatment in term animals (D19) results in constriction.^52^, ^53^, ^54^ Mid‐gestational (D11‐D15) treatment produced no phenotype.^53^ These results support initial clinical findings of PDA following administration of nonselective COX inhibitors as tocolytics in women at risk for preterm labor.^50^, ^51^, ^96^

Aminoglycoside antibiotics (gentamicin)^97^ and certain antacids, which inhibit cytochrome P450 enzymes (cimetidine)^98^ also cause PDA in mice. A recent cohort study confirmed the role of gentamicin in human PDA^99^ and cimetidine studies originated from human clinical observations.^98^, ^100^ Antibiotics, antacids, and COX inhibitors are routinely used in the treatment of pregnant women and neonates, emphasizing the utility of these animal models. Pharmacologic models also exist in other rodents, where vasodilatory mediators (PGE_2_, atrial natriuretic peptide, MgSO_4_, furosemide, phosphodiesterase 3 antagonists, endothelin receptor antagonists) or environmental perturbations (hypothermia, hypoxia, copper deficiency, LPS‐induced inflammation) result in PDA.

## 5 PDA IN HUMAN GENETIC SYNDROMES

Human PDA has a complicated and multi‐factorial genetic etiology.^101^ PDA likely exists as two overlapping disorders, with preterm PDA arising from prematurity, and term PDA from genetic alterations. Furthermore, PDA exists in syndromic and nonsyndromic forms, the former being more common in term PDA.^102^ A genetic basis for PDA is supported by (a) higher concordance rates of PDA in monozygotic vs dizygotic twins, (b) familial PDA with specific chromosomal deletions/mutations, (c) genetic polymorphisms conferring susceptibility to PDA, and (d) human dysmorphic syndromes with PDA and polygenic or monogenic inheritance.

**FIGURE 2:**
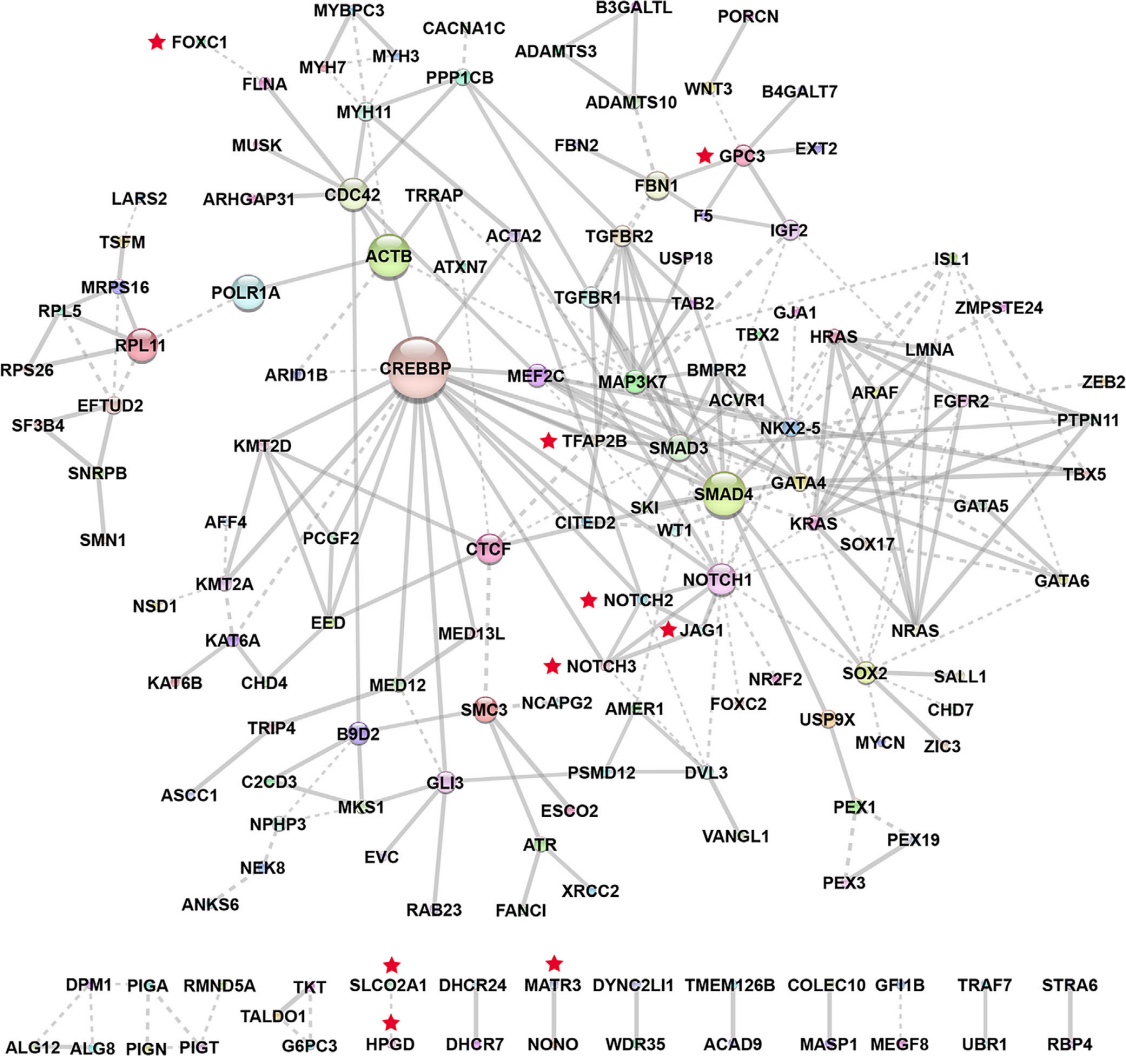
Protein‐protein interaction (PPI) network of effectors in PDA‐associated human single‐gene syndromes. Human single‐gene syndromes associated with PDA were used to construct a list of 224 potential effectors of DA function. This list was blindly assessed for known and predicted PPI including both direct (physical) and indirect (functional) associations using STRING 11.0. A minimum interaction score of 0.7 was selected representing a high confidence interval. The resulting network contains 219 proteins (nodes) and 256 interactions (edges) with a PPI enrichment *P* value of less than 1.0e‐16. Seventy‐one proteins were removed, as they lacked high confidence interactions. Edge thickness represents the confidence score of the PPI. Red stars indicate proteins with associated mouse models of PDA

**FIGURE 3:**
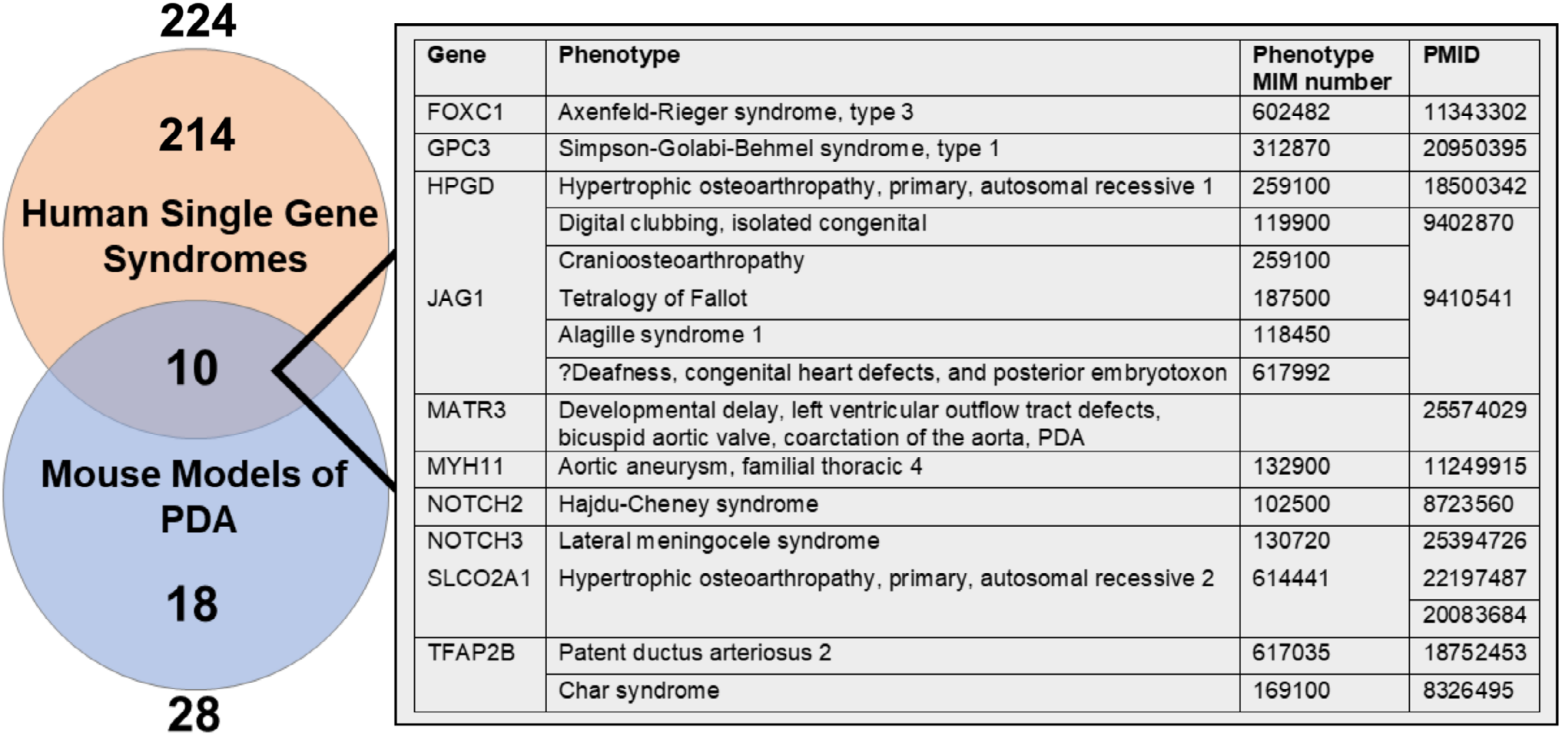
Overlap of mouse models of PDA with associated human single‐gene syndromes. Ten of the 28 identified mouse models of PDA were found to have correlated in the compiled list of 224 human single‐gene syndromes with PDA. The full table of human single‐gene syndromes with PDA is shown in Table S4

To better understand the genes crucial for DA development and function, we searched multiple databases for single‐gene syndromes associated with PDA. Using data from OMIM, GeneCards, Human Phenotype Ontology, DisGeNET, FindZebra, GeneReviews, and UniProtKB, a pooled list of n = 224 human single‐gene syndromes associated with PDA was generated (Table S2). PDA associations were verified through original sources (PMIDs provided). Deletion and duplication syndromes resulting in PDA were also compiled (Table S3). Two hundred and twenty‐four candidate effectors were assessed for protein‐protein interactions (PPI) using STRING V11.0. One hundred and forty‐eight proteins were identified as part of a high confidence PPI network (Figure 2). The use of a blind vote counting strategy between single‐gene syndromes and known mouse models revealed n = 10 genes associated with PDA in both mice and man (Figure 3, Table S4). This list contained several known PDA regulatory genes, including *HPGD*, *MYH11*, *JAG1‐NOTCH*, and *TFAP2B*. Due to irregular naming conventions and incomplete information on cross‐species orthologues, the molecular function of mouse and human PDA‐associated genes was compared. For top molecular function categories, 9/20 were common between mouse and human, suggesting higher levels of concordance than by gene name alone. A curated list of n = 41 human single‐gene syndromes consistently associated with PDA was derived from the GeneReviews database, to gauge the frequency of PDA in each syndrome (Table 1). Collectively, these data reveal similarities in the genetic landscape of PDA in mice and humans and identify pathways key for the regulation of fetal DA patency and postnatal closure.

**TABLE 1:** Genetic syndromes in GeneReviews citing PDA as a consistent clinical feature (n = 41 syndromes)

Syndrome	Gene(s)	Inheritance mode	PDA frequency
Char syndrome	*TFAP2B*	AD	High
Cantú syndrome	*ABCC9, KCNJ8*	AD	50%
Myhre syndrome	*SMAD4*	AD	20%
7q11.23 duplication syndrome	1.5‐ to 1.8‐Mb deletion in Williams‐Beuren syndrome critical region (*ELN*)	AD	15%‐21%
Mowat‐Wilson syndrome	*ZEB2*	AD	Moderate
Timothy syndrome	*CACNA1C*	AD	Moderate?
Loeys‐Dietz syndrome	*SMAD2*, *SMAD3*, *TGFB2*, *TGFB3*, *TGFBR1*, or *TGFBR2*	AD	Low
McKusick‐Kaufman syndrome	*MKKS*	AR	Low
3q29 recurrent deletion	hg38 chr3:195998129‐197623129	AD	12%
Heritable thoracic aortic aneurysms and dissections	*ACTA2*, *MYH11*,*TGFBR2*	AD	Variable
Warsaw syndrome	*DDX11*	AR	Low
X‐linked Opitz G/BBB syndrome	*MID1*	XL	Low
Weill‐Marchesani syndrome	*ADAMTS10*	AR	Low
*FLNA*‐related periventricular nodular heterotopia	*FLNA*	XL	Low
*MED12*‐related disorders	*MED12*	XL	Low
Roberts syndrome	*ESCO2*	AR	Low
Fanconi anemia	*FANCA,‐B,‐C,‐D2,‐E,‐F,‐G,‐I;BRCA2, BRIP1*	AR (mainly)	Low
Treacher Collins syndrome	*TCOF1* or *POLR1D*	AD (mainly)	Low
Phelan‐McDermid syndrome	22q13.3 deletion (SHANK3)	AD	Low
1q21.1 recurrent microdeletion	*1.35 Mb [hg36 @145‐146.35 Mb]*	AD	Low
*EZH2‐*related overgrowth	*EZH2*	AD	Low
Mandibulofacial dysostosis with microcephaly	*EFTUD2*	AD	Low
G6PC3 deficiency	*G6PC3*	AR	Low
Burn‐McKeown syndrome	*TXNL4A*	AR	Low
*SUCLG1*‐related mtDNA depletion	*SUCLG1*	AR	Low
*FBXL4*‐related mtDNA depletion syndrome	*FBXL4*	AR	Low
Xq28 duplication syndrome	0.5 Mb [Hg19@154.1 Mb to 154.6 Mb]	XL	Low
Simpson‐Golabi‐Behmel syndrome type 1	*GPC3*	XL	Low
Rubinstein‐Taybi syndrome	*CREBBP, EP300*	AD	Low
Heritable Pulmonary arterial hypertension	*BMPR2*	AD	Low
Feingold syndrome 1	*MYCN*	AD	Low
KAT6B disorders	*KAT6B*	AD	Low
Coffin‐Siris syndrome	*ARID1A*, *ARID1B*, *SMARCA4*, *SMARCB1*, *SMARCE1*, *SOX11*	AD	Low
Cranioectodermal dysplasia	*IFT122* (previously *WDR10*), *WDR35* (*IFT121*), *WDR19* (*IFT144*), or *IFT43* (previously *C14orf179*)	AR	Low
16p12.2 recurrent deletion	520‐kb @16p12.2	AD	Low
17q12 recurrent deletion syndrome	*1.4 Mb [*chr17: 34,815,072‐36,192,492*]*	AD	Low
*EED*‐related overgrowth	*EED*	AD	Low
Weiss‐Kruszka syndrome	*ZNF462*	AD	Low
Aymé‐Gripp syndrome	*MAF*	AD	Low
Emanuel syndrome	*duplication of 22q10‐22q11 and duplication of 11q23‐qter*	AD	Low
Sotos syndrome	*NSD1*	AD	Low

Abbreviation: PDA, patent ductus arteriosus.

## 6 DISCUSSION

PDA is a clinically relevant disorder of impaired circulatory adaptation to newborn life. Despite the knowledge of risk factors,^103^ current PDA treatment options are limited and the decision when or whether to treat remains an ongoing dilemma.^104^, ^105^ An understanding of the DA's complex molecular, environmental, and genetic regulation would benefit efforts to develop therapies, limit drug exposure, identify patients at risk for drug toxicity or treatment failure, and develop patient‐specific pharmacogenomic approaches. We recently conducted a transcriptomic meta‐analysis using published rodent microarrays and human preterm RNAseq data to identify candidate effectors involved in DA development and function.^43^ Although species and gestation‐stage differences of the data limited comparisons, 11 genes were found to be significantly up‐regulated in the DA compared to the aorta in both rodent and human tissues. Two genes, *PTGER4* and *TFAP2B*, have associated mouse models of PDA, supporting the notion that the correlation of human single‐gene syndromes and rodent models are useful for the study of PDA.

Prostaglandin signaling plays a key role in DA tone. COX‐1‐ and COX‐2‐derived PGE_2_ stimulates DA dilation through EP receptors, chief of which is EP_4_.^43^, ^106^, ^107^ This PGE_2_‐mediated dilation maintains DA patency throughout late gestation. Upon birth, the newly inflated lungs catabolize circulating PGE_2_ via HPGD. Decreased circulating PGE_2_ and oxygen‐stimulated constriction lead to the initial muscular DA closure shortly after birth.^108^ Additional studies implicate the PGE_2_‐EP_4_ axis in the remodeling of the fetal DA. EP_4_‐driven, adenylyl cyclase 6‐mediated^109^ hyaluronic acid deposition supports the migration of VSMCs from the media, through the elastic lamina, and into the subendothelial space to form intimal cushions, structures potentially key for DA closure in larger animals.^44^, ^47^ In addition, EP_4_‐driven EPAC1 activity promotes VSMC migration into the subendothelial space^110^ and an EP_4_‐mediated inhibition in elastogenesis and LOX expression contributes to remodeling.^44^, ^111^ Four mouse models of PDA target key prostaglandin signaling genes, highlighting this pathway's significance for DA development and function. Of note, the disruption of the prostaglandin pathway during late‐ but not mid‐gestation, in mice or humans, results in PDA, not premature constriction, contrary to expectations for the removal of a dilatory stimulus. This suggests a developmental programming role for the PGE_2_‐EP_4_, axis which warrants further investigation.

Monoallelic mutations in *TFAP2B* are associated with both Char syndrome‐associated PDA^20^, ^32^, ^78^ and single nucleotide mutation‐based nonsyndromic PDA.^79^, ^80^ TFAP2B likely regulates proliferation and differentiation during DA development, although the lack of defined downstream pathways and KO phenotypes makes this difficult to assess. *Tfap2b* expression is required for the expression of hypoxia‐inducible factor 2a (*Hif2a*) and endothelin‐1 (*Et‐1*). *Tfap2b* ^−/−^ animals also show decreased maturity in DA SMCs. Notably, *Tfap2b* is highly enriched in the DA vs aorta and was found significant by every rodent microarray in which it was assessed.^43^ TFAP2B's role in the differentiation of DA SMCs via HIF2A, ET‐1, and other downstream effectors requires further investigation to fully understand its contribution to DA development and function.

**FIGURE 4:**
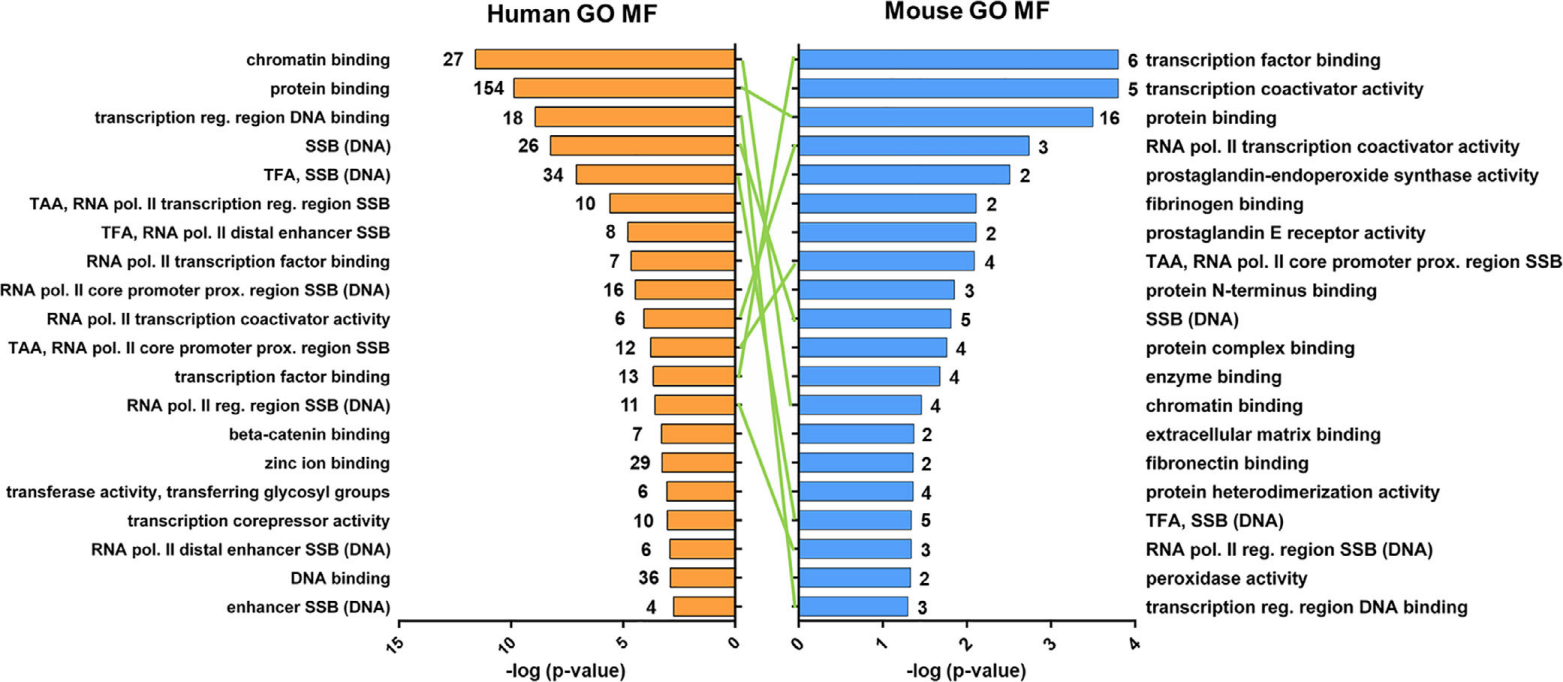
“Tornadogram” of top 20 GO molecular function (MF) terms common between known mouse models of patent ductus arteriosus (PDA) and human single‐gene syndromes with PDA. Genes known to be associated with PDA in mouse models (blue) and genes associated with PDA single‐gene syndromes in humans (orange) were categorized by GO MF (DAVID), plotted by *P* value, and compared across platforms. The number of genes represented in each category is displayed at the end of each bar. Like terms (n = 9) are connected by green lines

Currently, animal models are the primary means for studying DA regulatory mechanisms. Due to their well‐defined genetic composition, manageable size, short life span, ease of breeding, and litter size, mice are perhaps the most widely used of these models. To determine whether PDA‐associated genes in KO mice relate to human disease, we used online genetic databases to compile a comprehensive list of 224 single‐gene syndromes (Table S2) as well as 14 chromosomal deletions, duplications, or additions associated with PDA (Table S3). Of these 224 candidate effectors, 148 proteins were found to have high confidence PPI (Figure 2), suggesting these proteins may function as a coordinated network to regulate DA function. Several syndromes with associated mouse models such as Char syndrome (*TFAP2B*) and Alagille syndrome (*JAG1*) have well‐known associations with PDA. Conversely, *HPGD* and *NOTCH* genes (2 and 3) are more associated with PDA in mice. Only 10/28 known mouse models of PDA have human syndrome correlates (Figure 3), but 9 of those correlates showed high confidence PPI in our interaction network (Figure 2). Interspecies gene comparison is complicated by irregular naming conventions and orthologue conservation, which prevents direct comparisons. However, using functional annotation tools, we observed notable overlap between mouse models and human PDA syndromes in GO Biological Process (41.6%) (Figure 4), GO Cellular Component (37.5%), GO Molecular Function (48.0%), KEGG (66.7%), and UniProt (UP) Keywords (37.9%) (Table S5). While strong matches in GO Biological Process terms associated with heart and vascular development, patterning, or morphogenesis were expected, the number of GO terms related to RNA and DNA regulation coupled with “nucleoplasm” and “nucleus” lends more support to the idea that DA closure is a conserved, developmentally programmed event.

Although concordance between mouse and human PDA‐associated genes was modest (10/28) (Table S4), clinical data suggest the 18 noncorrelated mouse models may prove informative for human PDA. For example, while no known *PTGS* (1 or 2) or *PTGER4* mutations are associated with human PDA, the pharmacologic inhibition of COX enzymes in utero is linked to PDA in newborns.^50^, ^51^, ^96^, ^112^, ^113^ Similarly, while the mutations of platelet genes *Itga2b* and *Nfe2* confer PDA in mice but not humans, thrombocytopenia and various platelet indices correlate with PDA in preterm infants.^42^, ^114^ Information from some mouse models is even contradictory. While compound mutations in mouse *Gja5*;*Gja1* gap junction genes result in premature DA constriction, humans with *GJA1* mutations have PDA. Despite inconsistencies, deeper examination of candidate genes from mouse models will likely be informative for human PDA.

Our strategy comparing mouse KO models to human single‐gene syndromes with PDA has limitations. PDA may be polygenic or occur through epigenetic misregulation. Our search focused on the coding region mutations of single genes, but recent cardiovascular genetic studies suggest noncoding de novo variants may be important for CHD.^115^ PDA might also be secondary, resulting from the abnormal hemodynamics of complex CHD. Although enumerable human disorders have been modeled with KO mice, genetic dissimilarities exist in DA development between mice and humans.^43^ Screening strategies based on KO genes may overlook other single‐gene regulatory mechanisms. For example, Cantú syndrome patients, frequently affected by PDA, have monoallelic *activating* mutations in *ABCC9* or *KCNJ8*, which form K_ATP_ channels. Mouse models mimicking constitutive activation of *Abcc9* and *Kcnj8* have not been evaluated for PDA, although pharmacologic studies in mice correlate to the Cantú PDA phenotype.^116^ In other cases, a genetically defined PDA phenotype in humans may be overlooked in mice. *TBX1* mutations associated with 22q11 deletions and DiGeorge or velocardiofacial syndrome correlate with PDA. However, DA patency was not assessed in *Tbx1* KO mice despite the cyanosis and neonatal lethality common in mouse models of PDA.^117^ Of the 214 human single‐gene syndromes, which lack a corresponding mouse PDA model, 68 genes display embryonic lethality when deleted in mice and an additional 61 genes lack mouse models altogether. More importantly, mice with the targeted deletion of one of 26 genes corresponding to a human single‐gene syndrome exhibit neonatal lethality, consistent with PDA; however, their DA status was not reported. We also recognize that compensation for genetic mutations is species‐specific and genotype‐phenotype correlations may vary in mice and humans. For example, several mouse models lack PDA despite a corresponding human single‐gene syndrome with PDA, including ACTA2,^118^ MKKS,^119^ SLC25A24,^120^ and others. Furthermore, KO mice created to study PDA sometimes lack a phenotype, including KOs for endothelin ET‐A receptor,^121^ cytochrome P450 enzyme Cyp3a,^122^ PGE synthetic enzyme mPGES1,^123^ and Prx1 and Prx2 homeobox genes.^124^ Our comparisons may also suffer from the limiting nature of database searches. OMIM and other resources are not constantly curated, leading to possible omissions of human single‐gene syndromes associated with PDA that are too recent for inclusion, precluding an exhaustive compendium.

Our focus on KO mice is also complicated by strain‐selective modifiers, which alter penetrance or severity of some cardiovascular phenotypes,^125^ possibly including PDA. Furthermore, 4/28 mouse models of PDA had decreased phenotype severity on different backgrounds (*Ptger4* ^*−/−*^, *Itgα5* ^*−/−*^ *;Itgαv* ^*+/−*^, *Hand2* ^*−/−*^, *and Asxl2* ^*−/*−^). For unknown reasons, mice on C57Bl/6 backgrounds seem particularly susceptible to PDA phenotypes (Table S1). Many KO mice are never outcrossed to wholly different backgrounds, concealing potential PDA phenotypes. Finally, our comparison of mouse KO models to human single‐gene syndromes does not separate isolated PDA from PDA coexisting with complex CHD since our goal was a broad‐based and inclusive screen.

In addition to single‐gene syndromes, single nucleotide polymorphisms (SNPs) and single‐gene variants can indicate susceptibility to PDA. SNPs in TFAP2B, tumor necrosis factor (TNF) receptor associated factor 1 (TRAF1),^126^ angiotensin II receptor type 1 (AGTR1),^127^ elastin,^128^ methylenetetrahydrofolate reductase (MTHFR),^129^ and multiple other genes predispose infants to PDA. In addition, genetic variants in the CYP2C9 enzyme are associated with increased risk of indomethacin treatment failure in preterm neonates.^130^ SNPs, generally defined as occurring in >1% of the population, and rare variants occurring in <1% of the population certainly contribute to syndromic disorders, although distinctions between these are contentious and vary between populations.^131^ The involvement of SNPs and variants in PDA is a rapidly evolving field of research.^132^ While our search for single‐gene syndromes did identify several SNPs and rare variants, there is insufficient information to interpret their contributions to DA biology or pharmacogenomics^133^ at this time.

In summary, the expanding number of the mouse models of PDA, while not a perfect proxy for human vascular development, provides valuable information on vascular transition at birth and much‐needed research tools to study the mechanisms of DA development.^134^ Mouse models of PDA implicate important gene networks and multiple pathways that may be involved in human PDA. Comparison and functional analyses of mouse and human PDA‐associated genes will provide a better understanding of key regulatory steps that may serve as potential therapeutic targets for the management of PDA.

## CONFLICT OF INTEREST

The authors declare no conflicts of interest either personally or financially.

## AUTHOR CONTRIBUTIONS

**Michael Yarboro:** Conceptualization; data curation; funding acquisition; investigation; methodology; validation; visualization; writing ‐ original draft; writing‐review & editing. **Srirupa Gopal:** Data curation; formal analysis; methodology; writing ‐ original draft; writing‐review & editing. **Rachel Su:** Data curation; methodology; writing ‐ original draft. **Thomas Morgan:** Data curation; methodology; visualization; writing ‐ original draft. **Jeff Reese:** Conceptualization; data curation; formal analysis; funding acquisition; methodology; supervision; validation; visualization; writing ‐ original draft; writing‐review & editing.

## ETHICS STATEMENT

No human or animal subjects were recruited or used in the creation of this review.

## Supporting information


**Appendix** **S1**: Supplementary Information

## Data Availability

Supporting data are available in the article and online supplementary material.
