# Supplementary material for: Mouse models of patent ductus arteriosus (PDA) and their relevance for human PDA
Source: Dev Dyn. 2021 Aug 14;251(3):424–43. doi: 10.1002/dvdy.408 (PMC8814064; doi:10.1002/dvdy.408)
Supplement: Supplementary file 1 — Appendix S1: Supplementary Information [file DVDY-251-424-s001.docx]

**Supplement Material**

**Contents:**

1. Table S1. Genetic Models of PDA or *in utero* DA Closure in the Mouse
2. Table S2. Human Single Gene Syndromes Associated with PDA
3. Table S3. Chromosomal Deletions, Duplications, and Additions Associated with PDA in Humans
4. Table S4. Mouse Model Genes Associated with Single-Gene PDA Syndromes in Humans
5. Table S5. GO, KEGG, and UP Keywords Common Between Human PDA Syndromes and Mouse Models of PDA
6. Supplemental Methods

| **Table S1** | **Genetic Models of Patent Ductus Arteriosus (PDA) or *in utero* DA Closure in the Mouse (n=28)** | | | | | | |
| --- | --- | --- | --- | --- | --- | --- | --- |
| **Gene** | **Name** | **Year** | **PMID [Ref]** | **Phenotype** | **Secondary Phenotypes** | **Type** | **Background** |
| *Ptger4* | Prostanoid receptor EP4 | 1997 | 9363893 ^1^ | PDA and neonatal death | PDA associated pulmonary edema | Global constitutive KO | 129/SvEv |
|  |  | 1998 | 9600059 ^2^ | PDA and some neonatal death | PDA associated congestion of pulmonary arteries, shrunken disorganized alveolar structure, and dilation of left ventricle and pulmonary arteries | Global constitutive KO | 129/Ola + C57BL/6 mixed |
|  |  | 2004 | 15354288 ^3^ | PDA and neonatal death | PDA associated congestion of pulmonary arteries | Floxed Mouse | 129/SvEvTac + C57BL/6 mixed |
| *Ntf3* | Neurotrophin 3 | 1996 | 8841198 ^4^ | Premature DA closure *in utero* and at birth, and neonatal death (100%) | Atrial and ventricular septal defects, tetralogy of Fallot, valvular defects, general cardiac malformations | Global constitutive KO | C57BL/6 + CJ7 mixed |
| *Tfap2b* | Transcription factor AP2 beta | 1997 | 9271117 ^5^ | PDA and neonatal death noted by Gelb (unpublished) Ivey 2008 | Polycystic kidney disease which lead to apoptosis of the kidney epithelium | Global Constitutive KO | C57BL/6 |
|  |  | 2018 | 29804851 ^6^ | Delayed closure of the DA | Renal Malfunction | Global Constitutive KO (CRISPR) | C57Bl/6 |
| *Gja5/ Gja1* | Connexin 40/ Connexin 43 | 1999 | 10969038 ^7^ | Premature DA closure at E18.5 and neonatal death 100% | All embryos had persisting interventricular foramen, while many had persisting foramen primum and subcutaneous edema | Global constitutive KO | 129/Sv + C57BL/6 mixed |
| *Foxc1* | Forkhead box C1, Mesenchymal forkhead 1, Mesenchymal/mesodermal forkhead 1 | 1999 | 10479458 ^8^ | PDA and perinatal death | Interruptions and coarctations of the aortic arch, VSDs, valve defects, thin myocardium, prenatal death | Global constitutive double KO | 129 x BlackSwiss mixed ^9,10^ |
| *Ptgs1/ Ptgs2* dKO | Cyclooxygenase 1 / Cyclooxygenase 2 | 2000 | 10944235 ^11^ | PDA and neonatal death |  | Global constitutive KO | CD1 WT |
|  |  | 2001 | 11158594 ^12^ | PDA and 100% neonatal death in the dKO, partial phenotypes in the COX-2 -/-;COX-1 +/- |  | Global constitutive KO | 129/Ola + C57BL/6 mixed ^13,14^ |
|  |  | 2002 | 12189249 ^15^ |  |  | Global constitutive KO | 129/Ola + C57BL/6 mixed |
| *Ptgs1* | Cyclooxygenase 1 | 1995 | 8521478 ^13^ | No DA phenotype | Reduced indomethacin-induced gastric ulceration, reduced platelet aggregation and inflammatory response to arachidonic acid | Global constitutive KO | 129 + C57BL/6 mixed |
|  |  | 2006 | 16732282 ^16^ | PDA and some neonatal death | Defects in both renal and reproductive development | Global constitutive KO (loss of function) | 129S6/SvEvTac + C57BL/6 mixed |
| *Ptgs2* | Cyclooxygenase 2 | 2002 | 12189249 ^15^ | PDA or *in utero* constriction or depending on dosing regimen | Delayed birth (in Cox-1 KO) | Pharmacologic inhibition model | 129/Ola + C57BL/6 mixed |
| *Hpgd* | 15-Prostaglandin Dehydrogenase | 2002 | 11821873 ^17^ | PDA and neonatal death | PDA associated congestive heart failure | Global constitutive KO | C57BL/6 |
| *Myh11* | Myosin heavy chain 11 | 2000 | 10854329 ^18^ | Delayed DA closure (6h instead of 3h) | Generally defective SMC function resulting in failure to evacuate bladders, etc. | Global constitutive KO | C57BL/6 ^19^ |
| *Myocd* | Myocardin | 2008 | 18188448 ^20^ | PDA and neonatal death in 100% of KO mice | Dramatic decrease in SMC contractile potential and neural crest derived SMC differentiation | Conditional constitutive KO | SV129 + C57BL/6 mixed + described crosses ^21^ |
| *Gpc3* | Glypican 3 | 2009 | 19733558 ^22^ | PDA in a single replicate | Delayed coronary plexus formation and various cardiovascular defects including VSDs, common atrioventricular canal, double outlet right ventricle, and coronary artery fistulas | Global constitutive KO | C57Bl/6 |
| *Itga2b* | Integrin alpha 2b | 2010 | 19966813 ^23^ | PDA and neonatal death in 31% of KO mice | dysfunctional platelets and associated tendancy towards hemorrhage | Global constitutitve KO (loss of function) | C57BL/6J ^24,25^ |
| *Itga5/ Itgav* | Integrins alpha 5 and alpha v | 2010 | 20570943 ^26^ | PDA in 90% of KO mice surviving to 10-20 weeks and potential neonatal death of the remaining 50% of litters | Varying degrees of embryonic lethality dependent on genotype | Conditional constitutive KO | C57Bl/6 N7 or 129S4:C57Bl/6 |
| *Jag1* | Jagged 1 | 2010 | 21068062 ^27^ | PDA and neonatal death | Defects in contractile SMC differentiation in the DA and surrounding great arteries | Conditional constitutive KO | 129Sv + C57BL/6 mixed ^28,29^ |
| *Nfe2* | Nuclear factor, erythroid 2 | 2010 | 19966813 ^23^ | PDA and neonatal death in 70% of KO mice | PDA associated pulmonary hypertension, internal hemorrhage into peritoneal cavity, wall of urinary bladder, and frequently in GI tract, brain, testes, pericardium, and mouth | Global constitutitve KO | 129/Sv + C57BL/6 ^24,25^ |
| *Slco2a1* | Prostaglandin trasnporter | 2010 | 20083684 ^30^ | PDA and neonatal death in both KO and Hypomorph | PDA associated congestive heart failure | Global constitutitve KO | 129Sv + C57BL/6 mixed |
| *Smarca4* | SWI/SNF Brahma Brg1 | 2011 | 21518954 ^31^ | PDA and neonatal death in 30-40% of KO mice | Cardiovascular and intestinal defects relating to SMC gene expression and death | Conditional constitutive KO | Sv129 + C57BL/6 mixed |
| *Ilk* | Integrin linked kinase | 2011 | 21778429 ^32^ | PDA and neonatal death | Extreme dilated thoracic aortic aneurysms (~50% of abdominal cavity) | Conditional constitutive KO | SV129 + C57BL/6 mixed |
| *Ctnnb1* | Wnt-beta catenin | 2013 | 23382837 ^33^ | PDA and neonatal death | Melanoblasts are found in locations you would normally expect neural crest derived SMCs such as in the DA media, dilation of left atria, thrombus formation in dilated atrium | Conditional constitutive dominant positive | C57BL/6 ^33,34^ |
| *Hand2* | Heart and neural crest derivatives expressed protein 2 | 2013 | 23449628 ^35^ | PDA and neonatal death | VSD, skeletal hypoplasties, and various malformations of the digits | Global constitutive partial chromosomal duplication | C57Bl/10J |
| *Asxl2* | Additional sex combs like 2 | 2014 | 24860998 ^36^ | PDA and neonatal death | Low birth weight, thickened compact myocardium in the left ventricle, membranous ventricular septal defect, atrioventricular stenosis | Global constitutive KO | C57BL/6 |
| *Gdf2/ Bmp10* | Bone morphogenetic protein 9 and 10 | 2015 | 26056270 ^37^ | Reopening of the DA on P4 and subsequent death | Decrease in DA wall thickness and matrix deposition | Global constitutive KO with antibody treatment | C57BL6/J ^38^ |
| *Matr3* | Matrin 3 | 2015 | 25574029 ^39^ | PDA and neonatal death in 12% of heterzygotes | Homozygotes die preimplantation. Heterozygotes show cardiac defects including subaortic VSD and DORV, BAV, and CoA | Global constitutive KO | 129/SvJ + C57BL/6J or FVB/N + C57BL/6J |
| *Notch3/ Notch2* | Notch receptor 3 and 2 | 2015 | 26453897 ^40^ | PDA and neonatal death | Decrease in contractile gene expression and SMC development | Notch3 global constitutive KO, Notch2 conditional constitutive KO | C57BL/6 ^41,42^ |
| *Rbpj* | Recombination signal binding protein for immunoglobulin kappa J region | 2015 | 26742650 ^43^ | PDA and neonatal death | Decrease in contractile SMC gene expression | Conditional constitutive KO | C57BL/6 mixed ^44,45^ |
| *Lox* | Lysyl oxidase | 2017 | 28550176 ^46^ | Tortuosity in 100% of DAs, PDA in 22%, and neonatal death | Abnormal outflow tract formation, thoracic aortic aneurysm and dissection, ruptured diaphragms, impaired airways | Global constitutive KO | 129/SvJ + C57BL/6 mixed ^47^ |
| *Fbln1* | Fibulin 1 | 2020 | 32640908 ^48^ | PDA in 100% of KO mice | Hypoplastic intimal thickening of DA | Global constitutive KO | C57Bl/6 |

**Supplementary Table 1: Genetic Models of PDA or *in utero* DA Closure in the Mouse.**

Mouse models were identified by literature search. Gene names presented are official mouse gene symbol and may vary from names given in individual publications. Due to the emergent importance of strain to PDA phenotypes, strain information about each given mouse model was referenced.

| **Table S2** | **Human Single-Gene Syndromes Associated with PDA (n=224)** | | | | | | | |
| --- | --- | --- | --- | --- | --- | --- | --- | --- |
|  |  |  |  |  |  |  |  |  |
| **Gene/ Locus** | **Gene/Locus name** | **Gene/ Locus MIM number** | **Cytogenetic location** | **Phenotype** | **Phenotype MIM number** | **Inheritance pattern** | **Reference** | **PMID** |
| *ABCA3* | ATP-binding cassette-3 | 601615 | 16p13.3 | Surfactant metabolism dysfunction, pulmonary, 3 | 610921 | AR | Kunig et al. (2007) | 17719949 |
| *ABCC9* | ATP-binding cassette, subfamily C, member 9 (sulfonylurea receptor 2) | 601439 | 12p12.1 | Hypertrichotic osteochondrodysplasia (Cantu syndrome) | 239850 | AD | Harakalova et al (2012) | 22610116 |
|  |  |  |  |  |  |  | Scurr et al. (2011) | 21344641 |
| *ACAD9* | Acyl-CoA dehydrogenase family, member 9 | 611103 | 3q21.3 | Mitochondrial complex I deficiency, nuclear type 20 | 611126 | AR | Dewulf et al (2016) | 27233227 |
| *ACTA2* | Actin, alpha-2, smooth muscle, aorta | 102620 | 10q23.31 | Multisystemic smooth muscle dysfunction syndrome | 613834 | AD | Milewicz et al. (2010) | 20734336 |
|  |  |  |  | Moyamoya disease 5 | 614042 |  | Guo et al (2007) | 17994018 |
|  |  |  |  | Aortic aneurysm, familial thoracic 6 | 611788 | AD | Guo et al (2007) | 17994018 |
| *ACTB* | Actin, beta | 102630 | 7p22.1 | Baraitser-Winter syndrome 1 | 243310 | AD | Cuvertino et al (2017) | 29220674 |
|  |  |  |  |  |  |  | Verloes et al (2015) | 25052316 |
| *ACVR1* | Activin A Receptor, Type I | 102576 | 2q24.1 | Fibrodysplasia ossificans progressiva | 135100 | AD | Kaplan et al (2015) | 26097044 |
| *ADAMTS10* | A disintegrin-like and metalloproteinase with thrombospondin type 1 motif, 10 | 608990 | 19p13.2 | Weill-Marchesani syndrome 1, recessive | 277600 | AR | Faivre et al. (2003) | 14598350 |
| *ADAMTS3* | A Disintegrin-like and metalloproteinase with thrombospondin type 1 motif, 3 | 605011 | 4q13.3 | Hennekam lymphangiectasia-lymphedema syndrome 3 | 618154 | AR | Scheuerle et al (2018) | 30450763 |
| *ADAT3* | Adenosine deaminase, t-RNA- specific-3 | 615302 | 19p13.3 | Syndromic form of intellectual disability? | 615286 | AR | Thomas et al (2019) | 31687266 |
| *AFF4* | AF4/FMR2 family, member 4 | 604417 | 5q31.1 | CHOPS syndrome | 616368 | AD | Izumi et al. (2015) | 25730767 |
| *ALDH18A1* | Aldehyde dehydrogenase 18 family, member A1 (1-pyrroline-5-carboxylate synthetase) | 138250 | 10q24.1 | Cutis laxa, autosomal recessive, type IIIA | 219150 | AR | Fischer et al (2014) | 24913064 |
| *ALG12* | Dolichyl-P-mannose:Man-7-GlcNAc-2-PP-dolichyl-alpha-6- mannosyltransferase | 607144 | 22q13.33 | Congenital disorder of glycosylation, type Ig | 607143 | AR | Kranz et al. (2007) | 17506107 |
| *ALG8* | Alg8, S. cerevisiae, homolog of | 608103 | 11q14.1 | Congenital disorder of glycosylation, type Ih | 608104 | AR | Schollen et al. (2004) | 15235028 |
| *AMER1* | APC membrane recruitment protein 1 | 300647 | Xq11.2 | Osteopathia striata with cranial sclerosis | 300373 | XLD | Perdu et al (2011) | 20950377 |
| *AMMECR1* | Alport syndrome, mental retardation, midface hypoplasia, and elliptocytosis chromosomal region gene 1 | 300195 | Xq23 | Midface hypoplasia, hearing impairment, elliptocytosis, and nephrocalcinosis | 300990 | XLR | Basel-Vanagaite et al. (2017) | 28089922 |
| *ANKS6* | Ankyrin repeat and sterile alpha motif domains-containing protein 6 | 615370 | 9q22.33 | Nephronophthisis 16 | 615382 | AR | Hoff et al (2013) | 23793029 |
| *ARHGAP31* | Rho GTPase-activating protein 31 | 610911 | 3q13.32-q13.33 | Adams-Oliver syndrome 1 | 100300 | AD | Lin et al (1998) | 9823488 |
|  |  |  |  |  |  |  | Deeken and Caplan et al (1970) | 5536130 |
| *ARID1B* | AT-rich interaction domain-containing protein 1B | 614556 | 6q25.3 | Coffin-Siris syndrome 1 | 135900 | AD | Poyhonen et al (2004) | 15057123 |
|  |  |  |  |  |  |  | Kellermayer et al (2007) | 17523151 |
| *ARX* | Aristaless-related homeobox, X-linked | 300382 | Xp21.3 | Lissencephaly, X-linked 2 | 300215 | X-linked | Ogata et al (2000) | 10982975 |
|  |  |  |  | Hydranencephaly with abnormal genitalia |  |  |  |  |
| *ASCC1* | Activating signal cointegrator 1 complex, subunit 1 | 614215 | 10q22.1 | ?Spinal muscular atrophy with congenital bone fractures 2 | 616867 | AR | Knierim et al. (2016) | 26924529 |
| *ATP6V1E1* | ATPase, H+ transporting, V1 subunit E1 | 108746 | 22q11.21 | Cutis laxa, autosomal recessive, type IIC | 617402 | AR | Alazami et al (2016) | 27023906 |
| *ATR* | Ataxia-telangiectasia and Rad3-related (FRAP-related protein-1) | 601215 | 3q23 | Seckel syndrome 1 | 210600 | AR | Rappen et al (1993) | 8413337 |
|  |  |  |  | ?Cutaneous telangiectasia and cancer syndrome, familial | 614564 | AD |  |  |
| *ATXN7* | Ataxin 7 | 607640 | 3p14.1 | Spinocerebellar ataxia 7 | 164500 | AD | Whitney et al (2007) | 17254003 |
| *B3GLCT* | Beta 3-glucosyltransferase | 610308 | 13q12.3 | Peters-plus syndrome | 261540 | AR | Reis et al. (2008 | 18798333 |
| *B4GALT7* | Beta-1,4-galactosyltransferase 7 | 604327 | 5q35.3 | Ehlers-Danlos syndrome, spondylodysplastic type, 1 | 130070 | AR | Payet (1975) | 1221956 |
| *B9D2* | B9 domain-containing protein 2 | 611951 | 19q13.2 | Joubert syndrome 34 | 614175 | AR | Bachmann-Gagescu et al (2015) | 26092869 |
|  |  |  |  | ?Meckel syndrome 10 |  |  |  |  |
| *BCOR* | BCL6 corepressor | 300485 | Xp11.4 | Microphthalmia, syndromic 2 | 300166 | XLD | Hilton et al (2009) | 19367324 |
| *BMPR2* | Bone Morphogenetic Protein Receptor Type II | 600799 | 2q33.1-q33.2 | Pulmonary arterial hypertension, pulmonary vascular obstructive disease, PDA, atrial and ventricular septal defects, partial anomalous pulmonary venous return, transposition of the great arteries, atrioventricular canal, rare lesions with systemic to pulmonary shunt |  | AD | Roberts et al (2004) | 15358693 |
| *C12orf57* | Chromosome 12 open reading frame 57 | 615140 | 12p13.31 | Temtamy syndrome | 218340 | AR | Talisetti et al (2003) | 14564155 |
| *C2CD3* | C2 calcium-dependent domain-containing protein 3 | 615944 | 11q13.4 | Orofaciodigital syndrome XIV | 615948 | AR | Boczek et al (2018) | 30097616 |
| *CACNA1C* | Calcium channel, voltage-dependent, L type, alpha 1C subunit | 114205 | 12p13.33 | Timothy syndrome | 601005 | AD | Splawski et al. (2004) | 15454078 |
| *CCDC22* | Coiled-coil domain-containing protein 22 | 300859 | Xp11.23 | Ritscher-Schinzel syndrome 2 | 300963 | XLR | Voineagu et al. (2012) | 21826058 |
| *CD96* | CD96 antigen | 606037 | 3q13.1-q13.2 | C syndrome | 211750 | AD | Haaf et al. (1991) | 1746609 |
| *CDC42* | Cell division cycle 42 (GTP-binding protein, 25kD) | 116952 | 1p36.12 | Takenouchi-Kosaki syndrome | 616737 | AD | Takenouchi et al. (2015) | 26386261 |
|  |  |  |  |  |  |  | Martinelli et al. (2018) | 29394990 |
| *CDK10* | Cyclin-Dependent Kinase 10 | 603464 | 16q24.3 | Al Kaissi syndrome | 617694 | AR | Guen et al (2017) | 29130579 |
| *CDT1* | Chromatin licensing and DNA replication factor 1 | 605525 | 16q24.3 | Meier-Gorlin syndrome 4 | 613804 | AR | Guernsey et al (2011) | 21358631 |
| *CECR* | Cat eye syndrome | 115470 | 22q11 | Cat eye syndrome | 115470 | AD | Denavit et al (2004) | 15658620 |
| *CEP120* | Centrosomal protein, 120kD | 613446 | 5q13.2 | Short-rib thoracic dysplasia 13 with or without polydactyly | 616300 | AR | Shaheen et al (2015) | 25361962 |
| *CHD4* | Chromodomain helicase DNA-binding protein-4 | 603277 | 12p13.31 | Sifrim-Hitz-Weiss syndrome | 617159 | AD | Weiss et al (2016) | 27616479 |
| *CHD7* | Chromodomain helicase DNA binding protein 7 | 608892 | 8q12.2 | Hypogonadotropic hypogonadism 5 with or without anosmia | 612370 | AD | Jongmans et al. (2006) | 16155193 |
|  |  |  |  | CHARGE syndrome | 214800 | AD |  |  |
| *CHRM3* | Cholinergic receptor, muscarinic, 3 | 118494 | 1q43 | ?Prune belly syndrome | 100100 | AR | Yoshida et al (1995) | 7737585 |
| *CITED2* | CBP/p300-Interacting Transactivator, With GLU/ASP-Rich C-Terminal Domain, 2 | 602937 | 6q24.1 | Atrial septal defect 8, Ventricular septal defect 2 | 614433, 614431 | AD | Liu et al (2017) | 28687891 |
| *CLMP* | Coxsackievirus- and adenovirus receptor-like membrane protein | 611693 | 11q24.1 | Congenital short bowel syndrome | 615237 | AR | Hasosah et al. (2008) | 18209785 |
| *COL18A1* | Collagen XVIII, alpha-1 polypeptide | 120328 | 21q22.3 | Knobloch syndrome, type 1 | 267750 | AR | Wilson et al (1998) | 9677068 |
| *COLEC10* | Collectin 10 | 607620 | 8q24.12 | 3MC syndrome 3 | 248340 | AR | Chinen & Naritomi et al (1995) |  |
| *COQ4* | Coenzyme Q4, S. cerevisiae, homolog of | 612898 | 9q34.11 | Coenzyme Q10 deficiency, primary, 7 | 616276 | AR | Brea-Calvo et al. (2015) | 25658047 |
| *CREBBP* | CREB binding protein | 600140 | 16p13.3 | Rubinstein-Taybi syndrome 1 | 180849 | AD | Kanjilal et al. (1992) | 1404300 |
|  |  |  |  |  |  |  | Stevens and Bhakta (1995) | 8599359 |
| *CRELD1* | CYSTEINE-RICH PROTEIN WITH EGF-LIKE DOMAINS 1 | 607170 | 3p25.3 | Allelic variant-ATRIOVENTRICULAR SEPTAL DEFECT, SUSCEPTIBILITY TO, 2 | 606217 | AD | Maslen et al. (2006) | 17036335 |
| *CTCF* | CCCTC-binding factor | 604167 | 16q22.1 | Intellectual disability, autosomal dominant 21 | 615502 | AD | Gregor et al (2013) | 23746550 |
| *CTU2* | Cytosolic thiouridylase, subunit 2 | 617057 | 16q24.3 | Microcephaly, facial dysmorphism, renal agenesis, and ambiguous genitalia syndrome | 618142 | AR | Shaheen et al., 2016 | 27480277 |
| *CUX1* | Cut-like homeobox 1 | 116896 | 7q22.1 | Global developmental delay with or without impaired intellectual development | 618330 | AD | Platzer et al (2018) | 30014507 |
| *DER22t11-22* | Emanuel syndrome (supernumerary der(22)t(11;22) syndrome) | 609029 | 22q11.2 | Emanuel syndrome | 609029 | Inherited chromosomal imbalance | Carter et al (2009) | 19606488 |
| *DHCR24* | 24-dehydrocholesterol reductase | 606418 | 1p32.3 | Desmosterolosis | 602398 | AR | Andersson et al (2002) | 12457401 |
| *DHCR7* | Delta-7-dehydrocholesterol reductase | 602858 | 11q13.4 | Smith-Lemli-Opitz syndrome | 270400 | AR | Opitz et al., 1987 | 3322013 |
|  |  |  |  |  |  |  | Cunniff et al. 1997 | 9024557 |
|  |  |  |  |  |  |  | Kelley, 1998 | 9683618 |
| *DICER1* | Dicer, Drosophila, homolog of, 1 | 606241 | 14q32.13 | Pleuropulmonary blastoma | 601200 | AD | Foulkes et al (2011) | 21882293 |
|  |  |  |  |  |  |  |  |  |
| *DIP2B* | Disco-Interacting Protein 2 Homolog B | 611379 | 12q13.12 | Intellectual disability, FRA12A type | 136630 | AD | Berg et al (2000) | 10955484 |
| *DPM1* | Dolichyl-phosphate mannosyltransferase 1, catalytic subunit | 603503 | 20q13.13 | Congenital disorder of glycosylation, type Ie | 608799 | AR | Kim et al. (2000) | 10642597 |
| *DTNA* | Dystobrevin, alpha (dystrophin-related protein 3) | 601239 | 18q12.1 | Left ventricular noncompaction 1, with or without congenital heart defects | 604169 | AD | Ichida et al. (2001) | 11238270 |
| *DVL3* | Dishevelled 3 (homologous to Drosophila dsh) | 601368 | 3q27.1 | Robinow syndrome, autosomal dominant 3 | 616894 | AD | White et al. (2016) | 26924530 |
| *DYNC2LI1* | Dynein, cytoplasmic 2, light intermediate chain 1 | 617083 | 2p21 | Short-rib thoracic dysplasia 15 with polydactyly | 617088 | AR | Niceta et al. (2018) | 28857138 |
| *ECE1* | Endothelin converting enzyme 1 | 600423 | 1p36.12 | ?Hirschsprung disease, cardiac defects, and autonomic dysfunction | 613870 | AD | Hofstra et al. (1999) | 9915973 |
| *ECHS1* | Enoyl-CoA Hydratase, Short-Chain, 1, Mitochondrial | 602292 | 10q26.3 | Mitochondrial short-chain enoyl-CoA hydratase 1 deficiency | 616277 | AR | Nair et al (2016) | 27221955 |
| *EED* | Embryonic ectoderm development protein, mouse, homolog of | 605984 | 11q14.2 | Cohen-Gibson syndrome | 617561 | AD | Cooney et al. (2017) | 27868325 |
| *EFTUD2* | Elongation factor Tu GTP-binding domain-containing 2 | 603892 | 17q21.31 | Mandibulofacial dysostosis, Guion-Almeida type | 610536 | AD | Vincent et al. (2016) | 25790162 |
| *ELN* | Elastin | 130160 | 7q11.23 | Williams-Beuren syndrome |  | AD | Figueroa et al. (2008) | 18941598 |
| *EOGT* | EGF domain-specific O-linked N-acetylglucosamine transferase | 614789 | 3p14.1 | Adams-Oliver syndrome 4 | 615297 | AR | Shaheen et al (2013) | 23522784 |
| *ESCO2* | Establishment of cohesion 1, S. cerevisiae, homolog of, 2 | 609353 | 8p21.1 | Roberts syndrome | 268300 | AR | Goh et al. (2010) | 20101700 |
| *EVC* | EVC Ciliary Complex Subunit 1 | 604831 | 4p16.2 | Ellis-van Creveld syndrome | 225500 | AR | Vaughan et al. (2000) | 11376442 |
| *EXT2* | Exostosin Glycosyltransferase 2 | 608210 | 11p11.2 | Exostoses, multiple, type 2 | 133701 | AD | Gentile et al. (2019) | 30288735 |
|  |  |  |  | Seizures, scoliosis, and macrocephaly syndrome | 616682 | AR |  |  |
| *F5* | Coagulation factor V (proaccelerin, labile factor) | 612309 | 1q24.2 | Thrombophilia due to activated protein C resistance | 188055 | AD | Gorbe et al. (1999) | 10066036 |
| *FANCI* | FANCI gene | 611360 | 15q26.1 | Fanconi anemia, complementation group I | 609053 | AR | Savage et al (2015) | 26590883 |
| *FBN1* | Fibrillin-1 | 134797 | 15q21.1 | Weill-Marchesani syndrome 2, dominant | 608328 | AD | Faivre et al. (2003) | 14598350 |
| *FBN2* | Fibrillin-2 | 612570 | 5q23.3 | Contractural arachnodactyly, congenital | 121050 | AD | Viljoen et al (1994) | 7815423 |
| *FGFR2* | Fibroblast growth factor receptor-2 (bacteria-expressed kinase) | 176943 | 10q26.13 | Saethre-Chotzen syndrome | 101400 | AD | Okamoto et al (2016) | 30358290 |
| *FKBP14* | FK506-binding protein 14 | 614505 | 7p14.3 | Ehlers-Danlos syndrome, kyphoscoliotic type, 2 | 614557 | AR | Aldeeri et al. (2014) | 24773188 |
| *FLNA* | Filamin A, alpha (actin-binding protein-280) | 300017 | Xq28 | Congenital short bowel syndrome | 300048 | XLR | FitzPatrick et al. (1997) | 9279759 |
|  |  |  |  | Intestinal pseudoobstruction, neuronal | 300048 | XLR | FitzPatrick et al. (1997) | 9279759 |
|  |  |  |  | Heterotopia, periventricular, 1 | 300049 | XLD | Jefferies et al. (2010) | 20014127 |
| *FOXC1* | Forkhead, Drosophila, homolog-like 7 | 601090 | 6p25.3 | Axenfeld-Rieger syndrome, type 3 | 602482 | AD | Baruch and Erickson (2001) | 11343302 |
| *FOXC2* | Forkhead box C2 | 602402 | 16q24.1 | Lymphedema-distichiasis syndrome with renal disease and diabetes mellitus | 153400 | AD | Johnson et al. (1999) | 10086462 |
|  |  |  |  | Lymphedema-distichiasis syndrome |  |  |  |  |
| *FOXF1* | Forkhead box F1 | 601089 | 16q24.1 | Alveolar capillary dysplasia with misalignment of pulmonary veins | 265380 | AD | Sen et al (2004) | 15520767 |
|  |  |  |  |  |  |  | Vassal et al (1998) | 9475097 |
| *FTO* | Fat mass- and obesity-associated gene | 610966 | 16q12.2 | Growth delay, developmental delay, facial dysmorphism | 612938 | AR | Daoud et al. (2016) | 26378117 |
| *G6PC3* | Glucose-6-phosphatase, catalytic, 3 | 611045 | 17q21.31 | Neutropenia, severe congenital 4, autosomal recessive | 612541 | AR | Banka et al. (2011) | 20717171 |
|  |  |  |  | Dursun syndrome |  |  |  |  |
| *GATA4* | GATA-binding protein-4 | 600576 | 8p23.1 | Tetralogy of Fallot | 187500 | AD | Yang et al (2012) | 22101736 |
|  |  |  |  | Atrioventricular septal defect 4 | 614430 | AD |  |  |
|  |  |  |  | Atrial septal defect 2 | 607941 | AD |  |  |
|  |  |  |  | ?Testicular anomalies with or without congenital heart disease | 615542 | AD |  |  |
|  |  |  |  | Ventricular septal defect 1 | 614429 | AD |  |  |
| *GATA5* | GATA-binding protein 5 | 611496 | 20q13.33 | Congenital heart defects, multiple types, 5 | 617912 | AD; AR | Hempel et al (2017) | 28180938 |
| *GATA6* | GATA-binding protein-6 | 601656 | 18q11.2 | Persistent truncus arteriosus | 217095 |  | Kodo et al. (2009) | 19666519 |
|  |  |  |  | Pancreatic agenesis and congenital heart defects | 600001 | AD | Yorifuji et al. (1994) | 8071961 |
| *GFI1B* | Growth factor-independent 1B | 604383 | 9q34.13 | Bleeding disorder, platelet-type, 17 | 187900 | AD; AR | Ferreira et al (2017) | 28041820 |
| *GJA1* | Gap junction protein, alpha-1, 43kD (connexin 43) | 121014 | 6q22.31 | Hypoplastic left heart syndrome 1 | 241550 | AR | Brekke et al (1953) | 13050604 |
| *GLI3* | GLI-Kruppel family member GLI3 (oncogene GLI3) | 165240 | 7p14.1 | Pallister-Hall syndrome | 146510 | AD | Hall et al. (1980) | 7211952 |
| *GLIS3* | GLIS family zinc finger protein 3 | 610192 | 9p24.2 | Diabetes mellitus, neonatal, with congenital hypothyroidism | 610199 | AR | Dimitri et al (2015) | 26259131 |
| *GPC3* | Glypican 3 | 300037 | Xq26.2 | Simpson-Golabi-Behmel syndrome, type 1 | 312870 | XLR | Yano et al. (2011) | 20950395 |
| *HBHR* | Alpha-thalassemia/intellectual disability syndrome, type 1 | 141750 | 16pter-p13.3 | Alpha-thalassemia/intellectual disability syndrome, type 1 | 141750 | AD | Borochovitz et al. (1970) | 5433640 |
|  |  |  |  |  |  |  | Gibbons et al (1995) | 7726225 |
| *HCCS* | Holocytochrome c synthase (cytochrome c heme-lyase) | 300056 | Xp22.2 | Linear skin defects with multiple congenital anomalies 1 | 309801 | XLD | Prepeluh et al (2018) | 30068298 |
| *HPGD* | Hydroxyprostaglandin dehydrogenase 15-(NAD) | 601688 | 4q34.1 | Hypertrophic osteoarthropathy, primary, autosomal recessive 1 | 259100 | AR | Uppal et al (2008) | 18500342 |
|  |  |  |  | Digital clubbing, isolated congenital | 119900 | AR | Sinha et al. (1997) | 9402870 |
|  |  |  |  | Cranioosteoarthropathy | 259100 | AR |  |  |
| *HRAS* | Harvey rat sarcoma viral (v-Ha-ras) oncogene homolog | 190020 | 11p15.5 | Schimmelpenning-Feuerstein-Mims syndrome, somatic mosaic | 163200 |  | Rijntes-Jacobs et al (2010) | 20949522 |
| *IGBP1* | Immunoglobulin-binding protein 1 | 300139 | Xq13.1 | Corpus callosum, agenesis of, intellectual disability, ocular coloboma and micrognathia | 300472 | XLR | Graham et al (2003) | 14556245 |
| *IGF2* | Insulin-like growth factor-2, or somatomedin A | 147470 | 11p15.5 | ?Growth restriction, severe, with distinctive facies | 616489 | AD | Begemann et al. (2015) | 26154720 |
| *IRX5* | Iroquois homeo box protein 5 | 606195 | 16q12.2 | Hamamy syndrome | 611174 | AR | Bonnard et al. (2012) | 22581230 |
|  |  |  |  |  |  |  | Hamamy et al. (2007) | 17230486 |
| *ISL1* | Islet 1 | 600366 | 5q11.1 | PDA, ventricular septal defect |  | AD | Ma et al (2019) | 30390123 |
| *JAG1* | Jagged 1 | 601920 | 20p12.2 | Tetralogy of Fallot | 187500 | AD | Sánchez-Angulo et al (1997) | 9410541 |
|  |  |  |  | Alagille syndrome 1 | 118450 | AD |  |  |
|  |  |  |  | ?Deafness, congenital heart defects, and posterior embryotoxon | 617992 |  |  |  |
| *KAOGS* | Kagami-Ogata syndrome | 608149 | 14q32 | Kagami-Ogata syndrome | 608149 | AD | Sutton and Shaffer (2000) | 10951461 |
| *KAT6A* | K(lysine) acetyltransferase 6A | 601408 | 8p11.21 | Intellectual disability, autosomal dominant 32 | 616268 | AD | Tham et al. (2015) | 25728777 |
|  |  |  |  |  |  |  | Millan et al. (2016) | 27133397 |
| *KAT6B* | Lysine acetyltransferase 6B | 605880 | 10q22.2 | Genitopatellar syndrome | 606170 | AD | Brugha et al (2011) | 21412151 |
| *KCNH1* | Potassium voltage-gated channel, subfamily H, member 1 (ether-a-go-go, drosophila, homolog of) | 603305 | 1q32.2 | Zimmermann-Laband syndrome 1 | 135500 | AD | Robertson et al. (1998) | 9674908 |
| *KCNJ8* | Potassium inwardly rectifying channel subfamily J member 8 | 600935 | 12p12.1 | Hypertrichotic osteochondrodysplasia (Cantu syndrome) |  | AD | Grange et al. (2019) | 31828977 |
| *KMT2A* | Lysine-Specific Methyl Transferase 2A | 159555 | 11q23.3 | Wiedemann-Steiner syndrome | 605130 | AD | Min Ko et al (2016) | 27777327 |
| *KMT2D* | Lysine (K)-specific methyltransferase 2D | 602113 | 12q13.12 | Kabuki syndrome 1 | 147920 | AD | Niikawa et al (1988) | 3067577 |
| *KRAS* | Kirsten rat sarcoma-2 viral (v-Ki-ras2) oncogene homolog | 190070 | 12p12.1 | Schimmelpenning-Feuerstein-Mims syndrome, somatic mosaic | 163200 |  | Rijntes-Jacobs et al (2010) | 20949522 |
|  |  |  |  | Noonan syndrome 3 | 609942 | AD | Kratz et al. (2009) | 19396835 |
| *KYNU* | Kynureninase | 605197 | 2q22.2 | Vertebral, cardiac, renal, and limb defects syndrome 2 | 617661 | AR | Shi et al. (2017) | 28792876 |
| *LARS2* | Leucyl-tRNA synthetase, mitochondrial | 604544 | 3p21.31 | ?Hydrops, lactic acidosis, and sideroblastic anemia | 617021 | AR | Riley et al. (2016) | 26537577 |
| *LIFR* | Leukemia inhibitory factor receptor | 151443 | 5p13.1 | Stuve-Wiedemann syndrome/Schwartz-Jampel type 2 syndrome | 601559 | AR | Raas-Rothschild et al (2003) | 12910496 |
| *LMNA* | Lamin A/C | 150330 | 1q22 | Restrictive dermopathy, lethal | 275210 | AR | Bokenkamp et al. (2011) | 21915271 |
| *MAP3K7* | Mitogen-activated protein kinase kinase kinase 7 | 602614 | 6q15 | Frontometaphyseal dysplasia 2 | 617137 | AD | Morava et al. (2003) | 12503106 |
| *MASP1* | Mannan-binding lectin serine protease-1 (C4/C2 activating component of Ra-reactive factor) | 600521 | 3q27.3 | 3MC syndrome 1 | 257920 | AR | Rooryck et al., 2011 | 21258343 |
| *MATR3* | Matrin 3 | 164015 | 5q31.2 | Developmental delay, left ventricular outflow tract defects, bicuspid aortic valve, coarctation of the aorta, PDA |  | AD | Quintero-Rivera et al (2015) | 25574029 |
| *MECP2* | Methyl-CpG-binding protein-2 | 300005 | Xq28 | Intellectual disability, X-linked syndromic, Lubs type | 300260 | XLR | Belligni et al. (2010) | 20503343 |
| *MED12* | Mediator of RNA polymerase II transcription, subunit 12, S. cerevisiae, homolog of | 300188 | Xq13.1 | Opitz-Kaveggia syndrome | 305450 | XLR | Kato et al (1994) | 7802020 |
| *MED13L* | Mediator complex subunit 13-like | 608771 | 12q24.21 | Transposition of the great arteries, dextro-looped 1 | 608808 | AD | Asadollahi et al (2017) | 28645799 |
|  |  |  |  | Intellectual disability and distinctive facial features with or without cardiac defects | 616789 | AD |  |  |
| *MEGF8* | Multiple epidermal growth factor-like domains 8 | 604267 | 19q13.2 | Carpenter syndrome 2 | 614976 | AR | Twigg et al., 2012 | 23063620 |
| *MEF2C* | MADS Box Transcription Enhancer Factor 2, Polypeptide C | 600662 | 5q14.3 | Intellectual disability, stereotypic movements, epilepsy, and/or cerebral malformations | 613443 | AD | Qiao et al (2017) | 29104469 |
| *MID1* | Midline-1 | 300552 | Xp22.2 | Opitz GBBB syndrome, type I | 300000 | XLR | Winter et al (2003) | 12545276 |
| *MKKS* | McKusick-Kaufman syndrome gene | 604896 | 20p12.2 | Mckusick-kaufman Syndrome | 236700 | AR | Slavotinek et al (2015) | 20301675 |
| *MKS1* | MKS1 gene | 609883 | 17q22 | Meckel syndrome 1 | 249000 | AR | Salonen et al (1984) | 6486167 |
| *MRPS16* | Mitochondrial ribosomal protein S16 | 609204 | 10q22.2 | Combined oxidative phosphorylation deficiency 2 | 610498 | AR | Miller et al. (2004) | 15505824 |
| *MUSK* | Receptor tyrosine kinase MuSK | 601296 | 9q31.3 | Myasthenic syndrome, congenital, 9, associated with acetylcholine receptor deficiency | 616325 | AR | Maselli et al (2010) | 20371544 |
| *MYBPC3* | Myosin-Binding Protein C, Cardiac | 600958 | 11p11.2 | Cardiomyopathy, dilated, 1MM | 615396 | AD | Wessels et al (2014) | 25335496 |
| *MYCN* | Oncogene NMYC | 164840 | 2p24.3 | Feingold syndrome 1 | 164280 | AD | Frydman et al. (1997) | 9268091 |
| *MYH11* | Myosin, heavy polypeptide-11, smooth muscle | 160745 | 16p13.11 | Aortic aneurysm, familial thoracic 4 | 132900 | AD | Glancy et al (2001) | 11249915 |
| *MYH3* | Myosin, heavy polypeptide-3, skeletal muscle, embryonic | 160720 | 17p13.1 | Contractures, pterygia, and variable skeletal fusions syndrome 1A | 178110 | AD | Carapito et al. (2016) | 27381093 |
| *MYH7* | Myosin, Heavy Chain 7 | 160760 | 14q11.2 | Left ventricular noncompaction | 613426 | AD | Hirono et al (2020) | 32183154 |
| *MYRF* | Myelin regulatory factor | 608329 | 11q12.2 | Cardiac-urogenital syndrome | 618280 | AD | Pinz et al. (2018) | 29446546 |
| *NCAPG2* | Non-SMC condensin II complex subunit G2 | 608532 | 7q36.3 | Khan-Khan-Katsanis syndrome | 618460 | AR | Khan et al. (2019) | 30609410 |
| *NEK8* | Never in mitosis gene A-related kinase 8 | 609799 | 17q11.2 | ?Nephronophthisis 9 | 613824 |  | Rajagopalan et al (2016) | 26697755 |
|  |  |  |  | Renal-hepatic-pancreatic dysplasia 2 | 615415 | AR |  |  |
| *NFIX* | Nuclear factor I/X (CCAAT-binding transcription factor) | 164005 | 19p13.13 | Marshall-Smith syndrome | 602535 | AD | Shaw et al. (2010) | 16531739 |
| *NHS* | NHS gene | 300457 | Xp22.2-p22.1 | Cataract 40, X-linked | 302200 | X-linked | Coccia et al. (2009) | 19414485 |
| *NKX2-5* | NK2 homeobox-5 gene | 600584 | 5q35.1 | Hypoplastic left heart syndrome 2 | 614435 | AD | Brekke et al (1953) | 13050604 |
|  |  |  |  | Ventricular septal defect 3 | 614432 | AD | Peng et al. (2010) | 21110066 |
|  |  |  |  | Tetralogy of Fallot | 187500 | AD | Pauli et al (1999) | 10398271 |
| *NKX2-6* | NK2, Drosophila, homolog of, 6 | 611770 | 8p21.2 | Conotruncal heart malformations | 217095 |  | Kodo et al. (2009) | 19666519 |
|  |  |  |  | Persistent truncus arteriosus |  |  |  |  |
| *NMLFS* | Nablus mask-like facial syndrome (chromosome 8q22.1 deletion syndrome) | 608156 | 8q22.1 | Nablus mask-like facial syndrome | 608156 | AD | Barber et al. (2008) | 17940555 |
| *NONO* | NON-POU DOMAIN-CONTAINING OCTAMER-BINDING PROTEIN | 300084 | Xq13.1 | Intellectual disability, X-linked, syndromic 34 | 300967 | X linked | Scott et al (2017) | 27550220 |
| *NOTCH1* | Notch receptor 1 | 190198 | 9q34.3 | Aortic valve disease 1 | 109730 | AD | Stittrich et al (2014) | 25132448 |
|  |  |  |  | Adams-Oliver syndrome 5 | 616028 | AD |  |  |
| *NOTCH2* | Notch, Drosophila, homolog of, 2 | 600275 | 1p12 | Hajdu-Cheney syndrome | 102500 | AD | Rosser et al. (1996) | 8723560 |
| *NOTCH3* | Notch, Drosophila, homolog of, 3 | 600276 | 19p13.12 | Lateral meningocele syndrome | 130720 | AD | Gripp et al (2015) | 25394726 |
| *NPHP3* | Nephrocystin 3 | 608002 | 3q22.1 | Meckel syndrome 7 | 267010 | AR | Bergmann et al. (2008) | 18371931 |
|  |  |  |  | Renal-hepatic-pancreatic dysplasia 1 | 208540 | AR |  |  |
|  |  |  |  | Nephronophthisis 3 | 604387 | AR |  |  |
| *NR2F2* | Nuclear Receptor Sub-family 2, Group F, Member 2 | 107773 | 15q26.2 | Congenital heart defects, multiple types, 4 | 615779 | AD | Upadia et al (2018) | 29663647 |
| *NRAS* | Neuroblastoma RAS viral (v-ras) oncogene homolog | 164790 | 1p13.2 | Schimmelpenning-Feuerstein-Mims syndrome, somatic mosaic | 163200 |  | Rijntes-Jacobs et al (2010) | 20949522 |
| *NSD1* | Nuclear receptor binding SET domain protein 1 | 606681 | 5q35.3 | Leukemia, acute myeloid | 601626 | Somatic mutation; AD | Nagai et al (2005) | 12676901 |
|  |  |  |  | Sotos syndrome 1 | 117550 | AD | Kanemoto et al. (2006) | 16329110 |
| *PACS1* | Phosphofurin acidic cluster sorting protein 1 | 607492 | 11q13.1-q13.2 | Schuurs-Hoeijmakers syndrome | 615009 | AD | Martinez-Monseny et al. (2018) | 30113927 |
| *PEX1* | Peroxisome biogenesis factor-1 | 602136 | 7q21.2 | Peroxisome biogenesis disorder 1A (Zellweger) | 214100 | AR | Bowen et al (1964) | 14169466 |
| *PEX19* | Peroxisome biogenesis factor 19 (peroxisomal farnesylated protein) | 600279 | 1q23.2 | Peroxisome biogenesis disorder 12A (Zellweger) | 614886 | AR | Mohamed et al. (2010) | 20683989 |
| *PHGDH* | Phosphoglycerate dehydrogenase | 606879 | 1p12 | Neu-Laxova syndrome 1 | 256520 | AR | Manning et al (2004) | 14994231 |
| *PIGA* | Phosphatidylinositol glycan, class A | 311770 | Xp22.2 | Multiple congenital anomalies-hypotonia-seizures syndrome 2 | 300868 | XLR | Johnston et al (2012) | 22305531 |
| *PIGN* | Phosphatidylinositol glycan, class N | 606097 | 18q21.33 | Multiple congenital anomalies-hypotonia-seizures syndrome 1 | 614080 | AR | Maydan et al. (2011) | 21493957 |
| *PIGT* | Phosphatidylinositol glycan, class T | 610272 | 20q13.12 | Multiple congenital anomalies-hypotonia-seizures syndrome 3 | 615398 | AR | Nakashima et al. (2014) | 24906948 |
| *PKS* | Pallister-Killian syndrome | 601803 | 12p | Pallister-Killian syndrome | 601803 | Somatic mosaicism | Schinzel et al (1991) | 2002482 |
| *POLR1A* | Polymerase I, RNA, subunit A | 616404 | 2p11.2 | Acrofacial dysostosis, Cincinnati type | 616462 | AD | Weaver et al. (2015) | 25913037 |
| *PORCN* | Porcupine, Drosophila, homolog of | 300651 | Xp11.23 | Focal dermal hypoplasia | 305600 | XLD | Irvine et al (1996) | 8882775 |
| *POU1F1* | POU domain, class 1, transcription factor 1 (Pit1, growth hormone factor 1) | 173110 | 3p11.2 | Pituitary hormone deficiency, combined, 1 | 613038 | AD; AR | De Zegher et al. (1995) | 7593413 |
| *PPCS* | Phosphopantothenoylcysteine synthetase | 609853 | 1p34.2 | Cardiomyopathy, dilated, 2C | 618189 | AR | Iuso et al (2018) | 29754768 |
| *PPP1CB* | Protein phosphatase-1, catalytic subunit, beta isoform | 600590 | 2p23.2 | Noonan syndrome-like disorder with loose anagen hair 2 | 617506 | AD | Bertola et al. (2017) | 28211982 |
| *PRDM6* | PR domain-containing protein 6 | 616982 | 5q13.2 | Patent ductus arteriosus 3 | 617039 | AD | Lynch et al. (1965) | 5897316  27716515 |
| *PSMD12* | Proteasome 26S subunit, non-ATPase, 12 | 604450 | 17q24.2 | Stankiewicz-Isidor syndrome | 617516 | AD | Kury et al. (2017) | 28132691 |
| *PTPN11* | Protein tyrosine phosphatase, nonreceptor-type, 11 | 176876 | 12q24.13 | Noonan syndrome 1 | 163950 | AD | Noonan (1968) | 4386970 |
| *RAB23* | Ras-associated protein RAB23 | 606144 | 6p12.1-p11.2 | Carpenter syndrome | 201000 | AR | Alessandri et al. (2010) | 20358613 |
| *RBP4* | Retinol-binding protein-4, interstitial | 180250 | 10q23.33 | Retinal dystrophy, iris coloboma, and comedogenic acne syndrome | 615147 | AR | Cukras et al (2012) | 23189188 |
| *RMND5A* | Required For Meiotic Nuclear Division 5 Homolog A | 618964 | 2p11.2 | Giant occipitoparietal encephaloceles |  |  | Vogel et al (2012) | 22681319 |
| *RNF110* | Ring finger protein 110 (zinc finger protein-144) | 600346 | 17q12 | Turnpenny-Fry syndrome | 618371 | AD | Turnpenny et al., 2018 | 30343942 |
| *RPL11* | Ribosomal protein L11 | 604175 | 1p36.11 | Diamond-Blackfan anemia 7 | 612562 | AD | Gerrard et al (2013) | 23718193 |
| *RPL5* | Ribosomal protein L5 | 603634 | 1p22.1 | Diamond-Blackfan anemia 6 | 612561 | AD | Gazda et al (2008) | 19061985 |
| *RPS26* | Ribosomal protein S26 | 603701 | 12q13.2 | Diamond-Blackfan anemia 10 | 613309 | AD | Handler et al. (2009) | 19816270 |
| *SALL1* | Sal-like 1 | 602218 | 16q12.1 | Townes-Brocks syndrome 1 | 107480 | AD | Kohlhase et al (2007) | 20301618 |
|  |  |  |  | Townes-Brocks branchiootorenal-like syndrome |  |  |  |  |
| *SAMD9* | Sterile alpha motif domain-containing protein 9 | 610456 | 7q21.2 | MIRAGE syndrome | 617053 | AD | Narumi et al (2016) | 27182967 |
| *SEMA3E* | Semaphorin 3E | 608166 | 7q21.11 | ?CHARGE syndrome | 214800 | AD | Alazami et al. (2008) | 18553515 |
| *SF3B4* | Splicing factor 3B, subunit 4 | 605593 | 1q21.2 | Acrofacial dysostosis 1, Nager type | 154400 | AD | Petit et al. (2014) | 24003905 |
| *SH3PXD2B* | SH3 AND PX Domains-Containing Protein 2B | 613293 | 5q35.1 | Frank-ter Haar syndrome | 249420 | AR | Saeed et al (2011) | 21453629 |
| *SIK3* | Salt-inducible kinase 3 | 614776 | 11q23.3 | ?Spondyloepimetaphyseal dysplasia, Krakow type | 618162 | AR | Csukasi et al. (2018) | 30232230 |
| *SKI* | Avian sarcoma viral (v-ski) oncogene homolog | 164780 | 1p36.33-p36.32 | Shprintzen-Goldberg syndrome | 182212 | AD | Greally et al (1998) | 9508238 |
| *SLC25A24* | Solute carrier family 25 (mitochondrial carrier, phosphate carrier), member 24 | 608744 | 1p13.3 | Fontaine progeroid syndrome | 612289 | AD | Gorlin et al. (1960) | 13851313 |
| *SLC26A2* | Solute carrier family 26 (sulfate transporter), member 2 (diastrophic dysplasia sulfate transporter) | 606718 | 5q32 | Diastrophic dysplasia | 222600 | AR | De la Chapelle et al. (1972) | 4644462 |
|  |  |  |  | De la Chapelle dysplasia | 256050 | AR |  |  |
| *SLC29A3* | Solute carrier family 29 (nucleoside transporter), member 3 | 612373 | 10q22.1 | Histiocytosis-lymphadenopathy plus syndrome | 602782 | AR | Rossbach et al (2006) | 16155931 |
| *SLC35A3* | Solute Carrier Family 35 (UDP-N-Acetylglucosamine Transporter), Member 3 | 605632 | 1p21.2 | Arthrogryposis, intellectual disability, and seizures | 615553 | AR | Edmondson et al (2017) | 28777481 |
| *SLCO2A1* | Solute carrier organic anion transporter family, member 2A1 | 601460 | 3q22.1-q22.2 | Hypertrophic osteoarthropathy, primary, autosomal recessive 2 | 614441 | AR | Zhang et al (2012) | 22197487 |
|  |  |  |  |  |  |  | Chang et al (2010) | 20083684 |
| *SMAD3* | Mothers against decapentaplegic, Drosophila, homolog of, 3 | 603109 | 15q22.33 | Loeys-Dietz syndrome 3 | 613795 | AD | Van de Laar et al. (2012) | 22167769 |
| *SMAD4* | Mothers against decapentaplegic, Drosophila, homolog of, 4 | 600993 | 18q21.2 | Myhre syndrome | 139210 | AD | Caputo et al. (2012) | 22243968 |
| *SMC3* | Structural maintenance of chromosomes 3 | 606062 | 10q25.2 | Cornelia de Lange syndrome 3 | 610759 | AD | Gil-Rodríguez et al (2015) | 25655089 |
| *SMN1* | Survival of motor neuron 1, telomeric | 600354 | 5q13.2 | Spinal Muscle Atrophy 1 | 253300 | AR | Rudnik-Schoneborn et al (2008) | 18662980 |
| *SNRPB* | Small nuclear ribonucleoprotein polypeptides B and B1 | 182282 | 20p13 | Cerebrocostomandibular syndrome | 117650 | AD | Tooley et al (2016) | 26971886 |
| *SOX17* | SRY-box 17 | 610928 | 8q11.23 | Vesicoureteral reflux 3 | 613674 | AD | Gimelli et al (2010) | 20960469 |
| *SOX2* | SRY (sex determining region Y)-box 2 | 184429 | 3q26.33 | Optic nerve hypoplasia and abnormalities of the central nervous system | 206900 | AD | Bardakjian and Schneider (2005) | 15578584 |
|  |  |  |  | Microphthalmia, syndromic 3 |  |  |  |  |
| *SPECC1L* | Sperm antigen with calponin homology and coiled-coil domains 1-like | 614140 | 22q11.23 | Opitz GBBB syndrome, type II | 145410 | AD | Opitz et al. (1969) |  |
|  |  |  |  | Hypertelorism, Teebi type | 145420 | AD | Tsai et al (2002) | 12439902 |
| *STAMBP* | STAM binding protein | 606247 | 2p13.1 | Microcephaly-capillary malformation syndrome | 614261 | AR |  |  |
| *STRA6* | Stimulated by retinoic acid 6, mouse, homolog of | 610745 | 15q24.1 | Microphthalmia, syndromic 9 | 601186 | AR | Segel et al (2009) | 19839040 |
|  |  |  |  | Microphthalmia, isolated, with coloboma 8 |  |  | Pasutto et al. (2007) | 17273977 |
| *TAB2* | TAK1-Binding Protein 2 | 605101 | 6q25.1 | Congenital heart defects, nonsyndromic, 2 | 614980 | AD | Ackerman et al (2016) | 27452334 |
| *TALDO1* | Transaldolase-1 | 602063 | 11p15.5 | Transaldolase deficiency | 606003 | AR | Eyaid et al. (2013) | 23315216 |
| *TBC1D32* | TBC1 DOMAIN FAMILY, MEMBER 32 | 615867 | 6q22.31 | Allelic variant-VARIANT OF UNKNOWN SIGNIFICANCE |  |  | Adly et al. (2014) | 24285566 |
| *TBX1* | T-box 1 | 602054 | 22q11.21 | Velocardiofacial syndrome | 192430 | AD | McElhinney et al (2001) | 11731631 |
|  |  |  |  | Digeorge syndrome | 188400 | AD | Fukushima et al (1992) |  |
|  |  |  |  | Conotruncal anomaly face syndrome | 217095 |  | Matsuoka et al (1998) | 9737780 |
| *TBX2* | T-box 2 | 600747 | 17q23.2 | Vertebral anomalies and variable endocrine and T-cell dysfunction | 618223 | AD | Liu et al. (2018) | 29726930 |
| *TBX4* | T-Box Transcription Factor 4 | 601719 | 17q23.2 | Ischiocoxopodopatellar syndrome with or without pulmonary arterial hypertension | 147891 | AD | Galambos et al (2019) | 31151956 |
| *TBX5* | T-box 5 | 601620 | 12q24.21 | Holt-Oram syndrome | 142900 | AD | Glauser et al. (1989) | 2766565 |
| *TFAP2B* | Transcription factor AP-2 beta (activating enhancer-binding protein 2 beta) | 601601 | 6p12.3 | Patent ductus arteriosus 2 | 617035 | AD | Khetyar et al. (2008) | 18752453 |
|  |  |  |  | Char syndrome | 169100 | AD | Davidson (1993) | 8326495 |
| *TGFBR1* | Transforming growth factor, beta receptor I (activin A receptor type II-like kinase, 53kD) | 190181 | 9q22.33 | {Multiple self-healing squamous epithelioma, susceptibility to} | 132800 | AD | Loeys et al (2005) | 15731757 |
|  |  |  |  | Loeys-Dietz syndrome 1 | 609192 | AD | Sheikhzadeh et al. (2014) | 24344637 |
| *TGFBR2* | Transforming growth factor, beta receptor II, 70-80kD | 190182 | 3p24.1 | Loeys-Dietz syndrome 2 | 610168 | AD | Loeys et al. (2006) | 16928994 |
| *THOC6* | THO complex subunit 6 | 615403 | 16p13.3 | Beaulieu-Boycott-Innes syndrome | 613680 | AR | Boycott et al. (2010) | 20503307 |
| *TKT* | Transketolase | 606781 | 3p21.1 | Short stature, developmental delay, and congenital heart defects | 617044 | AR | Boyle et al. (2016) | 27259054 |
| *TMCO1* | Transmembrane and coiled-coil domains protein 1 | 614123 | 1q24.1 | cerebrofaciothoracic dysplasia | 213980 | AR | Cilliers et al. (2007) | 17351359 |
| *TMEM126B* | Transmembrane protein 126B | 615533 | 11q14.1 | Mitochondrial complex I deficiency, nuclear type 29 | 618250 | AR | Alston et al (2016) | 27374774 |
| *TMEM94* | Transmembrane protein 94 | 618163 | 17q25.1 | Intellectual developmental disorder with cardiac defects and dysmorphic facies | 618316 | AR | Stephen et al. (2018) | 30526868 |
| *TP63* | Tumor protein p63 (tumor protein p73-like) | 603273 | 3q28 | Hay-Wells syndrome | 106260 | AD | Sutton et al. (2009) | 19676059 |
| *TRAF7* | TNF receptor-associated factor 7 | 606692 | 16p13.3 | Cardiac, facial, and digital anomalies with developmental delay | 618164 | AD | Tokita et al. (2018) | 29961569 |
| *TRIP4* | Thyroid hormone receptor interactor 4 | 604501 | 15q22.31 | Spinal muscular atrophy with congenital bone fractures 1 | 616866 | AR | Knierim et al. (2016) | 26924529 |
| *TRRAP* | Transformation/transcription domain-associated protein | 603015 | 7q22.1 | Developmental delay with or without dysmorphic facies and autism | 618454 | AD | Cogne et al. (2019) | 30827496 |
| *TSFM* | Ts translation elongation factor, mitochondrial | 604723 | 12q14.1 | Combined oxidative phosphorylation deficiency 3 | 610505 | AR | Smeitink et al. (2006) | 17033963 |
| *UBR1* | Ubiquitin-Protein Ligase E3 Component N-Recognin 1 | 605981 | 15q15.2 | Johanson-Blizzard syndrome | 243800 | AR | Fallahi et al (2011) | 20556423 |
| *USP18* | Ubiquitin-specific protease 18 | 607057 | 22q11.21 | Pseudo-TORCH syndrome 2 | 617397 | AR | Meuwissen et al., 2016 | 27325888 |
| *USP9X* | Ubiquitin-specific protease-9, X chromosome (Drosophila fat facets related, X-linked) | 300072 | Xp11.4 | Intellectual disability, X-linked 99, syndromic, female-restricted | 300968 | XLD | Reijnders et al. (2016) | 26833328 |
| *VANGL1* | Vang-like 1 | 610132 | 1p13.1 | Caudal regression syndrome | 600145 | AD | Finer et al (1978) | 657575 |
| *VPS33A* | Vacuolar protein sorting 33, yeast, homolog of, A | 610034 | 12q24.31 | Mucopolysaccharidosis-plus syndrome | 617303 | AR | Kondo et al. (2017) | 28013294 |
| *WAC* | WW domain-containing adaptor with coiled-coil region | 615049 | 10p12.1 | Desanto-Shinawi syndrome | 616708 | AD | Wentzel et al (2011) | 21522184 |
| *WDR35* | WD repeat-containing protein 35 | 613602 | 2p24.1 | Cranioectodermal dysplasia 2 | 613610 | AR | Bacino et al. (2012) | 22987818 |
| *WNT3* | Wingless-type MMTV integration site family, member 3 | 165330 | 17q21.31-q21.32 | ?Tetra-amelia syndrome 1 | 273395 | AR | Zimmer et al (1985) | 4076260 |
| *WSHC5* | WASH complex, subunit 5 | 610657 | 8q24.13 | Ritscher-Schinzel syndrome 1 | 220210 | AR | Leonardi et al (2001) | 11484200 |
| *WT1* | WT1 Transcription Factor | 607102 | 11p13 | Ambiguous genitalia with absence of gonadal dysgenesis and kidney disease |  |  | Köhler et al (2004) | 15191353 |
| *XRCC2* | X-ray repair, complementing defective, repair in Chinese hamster cells-2 | 600375 | 7q36.1 | ?Fanconi anemia, complementation group U | 617247 | AR | Shamseldin et al. (2012) | 22232082 |
| *YY1AP1* | YY1 associated protein 1 | 607860 | 1q22 | Grange syndrome | 602531 | AR | Grange et al (1998) | 9489789 |
| *ZEB2* | Zinc finger E box-binding homeobox 2 | 605802 | 2q22.3 | Mowat-Wilson syndrome | 235730 | AD | Wakamatsu et al. (2001) | 11279515 |
|  |  |  |  |  |  |  | Strenge et al. (2007) | 17567886 |
| *ZIC3* | Zic family, member 3 | 300265 | Xq26.3 | Heterotaxy, visceral, 1, X-linked | 306955 | XLR | Mathias et al. (1987) | 3674105 |
| *ZNF148* | Zinc finger protein-148 | 601897 | 3q21.2 | Global developmental delay, absent or hypoplastic corpus callosum, and dysmorphic facies | 617260 | AD | Stevens et al. (2016) | 27964749 |

Brackets, "[ ]", indicate "nondiseases," mainly genetic variations that lead to apparently abnormal laboratory test values (e.g., dysalbuminemic euthyroidal hyperthyroxinemia). Braces, "{ }", indicate mutations that contribute to susceptibility to multifactorial disorders (e.g., diabetes, asthma) or to susceptibility to infection (e.g., malaria). A question mark, "?", before the phenotype name indicates that the relationship between the phenotype and gene is provisional. More details about this relationship are provided in the comment field of the map and in the gene and phenotype OMIM entries.

**Supplementary Table 2**. **Human single-gene syndromes associated with PDA**

The OMIM database was searched for genes which complied with three major criteria: association with a single-gene syndrome, a database association with PDA, and available references linking to a human PDA phenotype. OMIM searches were limited to single-gene syndromes and made use of both the ‘and’ command and exhaustive combinations of the terms ‘patent,’ ‘ductus,’ ‘arteriosus,’ ‘arterial duct’, and ‘Botalli”. References were validated or compiled manually. Additional online resources including GeneCards, Human Phenotype Ontology, DisGeNET, FindZebra, GeneReviews and UniProtKB were then used to validate the exhaustive nature of the OMIM list with novel genes added. Some genes associated with PDA in genetic syndrome databases (Table 1) could not be verified by primary sources (e.g. DDX11, EZH2, TXNL4A, SUCLG1, ZNF462, MAF, and others). These examples may represent clinical syndromes associated with multiple genes where only one gene is clearly linked to PDA, or syndromes belonging to a class of disorders where PDA is a feature of one specific gene and genotype-phenotype association. This list is limited by the databases used to assemble it and should not be considered exhaustive.

| **Table S3** | **Chromosomal Deletions, Duplications, and Additions Associated with PDA in the Human (N=15)** | | | | | | | |
| --- | --- | --- | --- | --- | --- | --- | --- | --- |
|  |  | | | | | | | |
| **Gene/ Locus** | **Gene/ Locus name** | **Gene/ Locus MIM number** | **Cytogenetic location** | **Phenotype** | **Phenotype MIM number** | **Inheritance** | **Reference** | **PMID** |
| DEL10q26 | Chromosome 10q26 deletion syndrome | 609625 | 10q26 | Chromosome 10q26 deletion syndrome | 609625 | AD | Tanabe et al. (1999) | 10530074 |
|  |  |  |  |  |  |  | Yatsenko et al. (2009) | 19558528 |
| DEL14q11q22 | Chromosome 14q11-q22 deletion syndrome | 613457 | 14q11-q22 | Chromosome 14q11-q22 deletion syndrome | 613457 | Isolated cases | Shapira et al. (1994) | 7977460 |
|  |  |  |  |  |  |  | Zahir et al. (2007) | 17545556 |
| DEL17q23.1q23.2 | Chromosome 17q23.1-q23.2 deletion syndrome | 613355 | 17q23.1-q23.2 | Chromosome 17q23.1-q23.2 deletion syndrome | 613355 | Isolated cases | Ballif et al. (2010) | 20206336 |
| DEL18q | Chromosome 18q deletion syndrome | 601808 | 18q | Chromosome 18q deletion syndrome | 601808 | AD | Versacci et al. (2005) | 16100728 |
| DEL1q41q42 | Chromosome 1q41-q42 deletion syndrome | 612530 | 1q41-q42 | Chromosome 1q41-q42 deletion syndrome | 612530 | Isolated cases | Filges et al. (2010) | 20358614 |
| DEL22q11.2 | Chromosome 22q11.2 deletion syndrome, distal | 611867 | 22q11.2 | Chromosome 22q11.2 deletion syndrome, distal | 611867 | PH | Rauch et al (2005) | 15831592 |
| DEL3pterp25 | 3p- syndrome (chromosome 3pter-p25 deletion syndrome) | 613792 | 3pter-p25 | 3p- syndrome | 613792 | AD | Nienhaus et al (1992) | 1481811 |
| DEL3q29 | Chromosome 3q29 microdeletion syndrome | 609425 | 3q29 | Chromosome 3q29 microdeletion syndrome | 609425 | Isolated cases | Li et al (2009) | 19460468 |
| DEL6pter | Chromosome 6pter-p24 deletion syndrome | 612582 | 6pter-p24 | Chromosome 6pter-p24 deletion syndrome | 612582 | Isolated cases | DeScipio et al. (2005) | 15704124 |
| DEL6q24q25 | Chromosome 6q24-q25 deletion syndrome | 612863 | 6q24-q25 | Chromosome 6q25-q25 deletion syndrome | 612863 | PH | Caselli et al (2007) | 17512813 |
| DEL8q13 | Mesomelia-synostoses syndrome (Chromosome 8q13 deletion syndrome) | 600383 | 8q13 | Mesomelia-synostoses syndrome | 600383 | AD | Day-Salvatore & McLean et al (1998) | 9856555 |
| DEL9p | Chromosome 9p deletion syndrome | 158170 | 9p | Chromosome 9p deletion syndrome | 158170 | AD | Alfi et al. (1973) | 4541805 |
|  |  |  |  |  |  |  | Swinkels et al. (2008) | 18452192 |
| DER22t11-22 | Emanuel syndrome (supernumerary der(22)t(11;22) syndrome) | 609029 | 22q11.2 | Emanuel syndrome | 609029 | Inherited chromosomal imbalance | Carter et al (2009) | 19606488 |
| DUP7q11.23 | Chromosome 7q11.23 duplication syndrome | 609757 | 7q11.23 | Chromosome 7q11.23 duplication syndrome | 609757 | AD | Van der Aa et al. (2009) | 19249392 |
| EYA4 | Eyes Absent 4 | 603550 | 6q23.2-q24.1 | Microcephaly, short stature, PDA, sensorineural hearing loss |  | AD | Dutrannoy et al (2009) | 19576303 |

**Supplementary Table 3**. **Chromosomal deletions, duplications, and additions associated with PDA in the human**

The OMIM database was searched for genes which complied with three major criteria: association with a single-gene syndrome, a database association with PDA, and available references linking to a human PDA phenotype. OMIM searches were limited to single-gene syndromes and made use of both the ‘and’ command and exhaustive combinations of the terms ‘patent,’ ‘ductus,’ ‘arteriosus,’ ‘arterial duct’, and ‘Botalli”. References were validated or compiled manually. Additional online resources including GeneCards, Human Phenotype Ontology, DisGeNET, FindZebra, GeneReviews and UniProtKB were then used to validate the exhaustive nature of the OMIM list with novel genes added.

| **Table S4** | **Mouse Model Genes Associated with Single-Gene PDA Syndromes in Humans (n=10 Genes)** | | | | | | | |
| --- | --- | --- | --- | --- | --- | --- | --- | --- |
|  |  |  |  |  |  |  |  |  |
|  |  |  |  |  |  |  |  |  |
| **Gene/ Locus** | **Gene/Locus name** | **Gene/ Locus MIM number** | **Cytogenetic location** | **Phenotype** | **Phenotype MIM number** | **Inheritance** | **Reference** | **PMID** |
| *FOXC1* | Forkhead, Drosophila, homolog-like 7 | 601090 | 6p25.3 | Axenfeld-Rieger syndrome, type 3 | 602482 | AD | Baruch and Erickson (2001) | 11343302 |
| *GJA1** | Gap junction protein, alpha-1, 43kD (connexin 43) | 121014 | 6q22.31 | Hypoplastic left heart syndrome 1 | 241550 | AR | Brekke et al (1953) | 13050604 |
| *GPC3* | Glypican 3 | 300037 | Xq26.2 | Simpson-Golabi-Behmel syndrome, type 1 | 312870 | XLR | Yano et al. (2011) | 20950395 |
| *HPGD* | Hydroxyprostaglandin dehydrogenase 15-(NAD) | 601688 | 4q34.1 | Hypertrophic osteoarthropathy, primary, autosomal recessive 1 | 259100 | AR | Uppal et al (2008) | 18500342 |
|  |  |  |  | Digital clubbing, isolated congenital | 119900 | AR | Sinha et al. (1997) | 9402870 |
|  |  |  |  | Cranioosteoarthropathy | 259100 | AR |  |  |
| *JAG1* | Jagged 1 | 601920 | 20p12.2 | Tetralogy of Fallot | 187500 | AD | Sánchez-Angulo et al (1997) | 9410541 |
|  |  |  |  | Alagille syndrome 1 | 118450 | AD |  |  |
|  |  |  |  | ?Deafness, congenital heart defects, and posterior embryotoxon | 617992 |  |  |  |
| *MATR3* | Matrin 3 | 164015 | 5q31.2 | Developmental delay, left ventricular outflow tract defects, bicuspid aortic valve, coarctation of the aorta, PDA |  | AD | Quintero-Rivera et al (2015) | 25574029 |
| *MYH11* | Myosin, heavy polypeptide-11, smooth muscle | 160745 | 16p13.11 | Aortic aneurysm, familial thoracic 4 | 132900 | AD | Glancy et al (2001) | 11249915 |
| *NOTCH2* | Notch, Drosophila, homolog of, 2 | 600275 | 1p12 | Hajdu-Cheney syndrome | 102500 | AD | Rosser et al. (1996) | 8723560 |
| *NOTCH3* | Notch, Drosophila, homolog of, 3 | 600276 | 19p13.12 | Lateral meningocele syndrome | 130720 | AD | Gripp et al (2015) | 25394726 |
| *SLCO2A1* | Solute carrier organic anion transporter family, member 2A1 | 601460 | 3q22.1-q22.2 | Hypertrophic osteoarthropathy, primary, autosomal recessive 2 | 614441 | AR | Zhang et al (2012) | 22197487 |
|  |  |  |  |  |  |  | Chang et al (2010) | 20083684 |
| *TFAP2B* | Transcription factor AP-2 beta (activating enhancer-binding protein 2 beta) | 601601 | 6p12.3 | Char syndrome | 169100 | AD | Khetyar et al. (2008) | 18752453 |
|  |  |  |  |  |  |  | Davidson (1993) | 8326495 |

*While a mouse model does exist for this gene, it is not of PDA, but premature DA closure. This gene was included as it is likely still relevant for both human and mouse DA biology

**Supplementary Table 4: Mouse Model Genes Associated with Single-Gene PDA Syndromes in Humans.**

Our list of genetic mouse models of PDA was compared to our OMIM generated list of human single gene syndromes associated with PDA. This resulted in the identification of 10 mouse models of PDA which have associated single-gene syndromes in humans. Additionally, one gene was found to have a human single-gene syndrome associated with PDA and a mouse model in which the DA closes prematurely *in utero.*

| **Table S5** | **GO, KEGG, and UP Keywords Common Between Human PDA Syndromes and Mouse Models of PDA** | | | | |
| --- | --- | --- | --- | --- | --- |
|  | |  |  |  |  |
|  | | Human | Human | Mouse | Mouse |
| **GO Biological Process - Overlap: 57/137 Common, 41.6%** | | **Count^1^** | **p Value** | **Count** | **p Value** |
| GO:0000122~negative regulation of transcription from RNA polymerase II promoter | | 41 | 2.01E-15 | 8 | 7.37E-05 |
| GO:0001568~blood vessel development | | 6 | 1.12E-04 | 4 | 1.59E-04 |
| GO:0001569~patterning of blood vessels | | 4 | 5.22E-03 | 2 | 5.81E-02 |
| GO:0001570~vasculogenesis | | 6 | 7.08E-04 | 4 | 1.39E-04 |
| GO:0001658~branching involved in ureteric bud morphogenesis | | 5 | 1.93E-03 | 3 | 2.32E-03 |
| GO:0001701~in utero embryonic development | | 16 | 1.63E-08 | 5 | 9.52E-04 |
| GO:0001756~somitogenesis | | 5 | 1.46E-03 | 2 | 8.04E-02 |
| GO:0001822~kidney development | | 7 | 7.85E-04 | 4 | 9.36E-04 |
| GO:0001947~heart looping | | 14 | 5.86E-13 | 3 | 3.84E-03 |
| GO:0001974~blood vessel remodeling | | 7 | 2.67E-06 | 3 | 1.87E-03 |
| GO:0003007~heart morphogenesis | | 4 | 7.61E-03 | 3 | 4.09E-03 |
| GO:0003151~outflow tract morphogenesis | | 9 | 1.01E-07 | 3 | 2.93E-03 |
| GO:0003184~pulmonary valve morphogenesis | | 4 | 2.26E-04 | 2 | 1.63E-02 |
| GO:0003281~ventricular septum development | | 4 | 5.22E-03 | 2 | 5.53E-02 |
| GO:0006351~transcription, DNA-templated | | 43 | 4.12E-04 | 11 | 2.35E-04 |
| GO:0006355~regulation of transcription, DNA-templated | | 33 | 2.56E-03 | 11 | 1.09E-03 |
| GO:0006357~regulation of transcription from RNA polymerase II promoter | | 12 | 2.54E-02 | 5 | 2.69E-03 |
| GO:0007219~Notch signaling pathway | | 10 | 1.58E-05 | 5 | 3.38E-05 |
| GO:0007507~heart development | | 30 | 1.10E-23 | 9 | 2.96E-09 |
| GO:0007512~adult heart development | | 4 | 5.24E-04 | 3 | 2.54E-04 |
| GO:0008284~positive regulation of cell proliferation | | 19 | 2.88E-05 | 10 | 5.29E-08 |
| GO:0008285~negative regulation of cell proliferation | | 14 | 1.68E-03 | 8 | 1.14E-06 |
| GO:0009887~organ morphogenesis | | 7 | 1.12E-03 | 3 | 1.15E-02 |
| GO:0009948~anterior/posterior axis specification | | 3 | 1.92E-02 | 2 | 3.24E-02 |
| GO:0010468~regulation of gene expression | | 5 | 3.85E-02 | 3 | 7.68E-02 |
| GO:0010628~positive regulation of gene expression | | 14 | 3.11E-05 | 7 | 2.22E-05 |
| GO:0010862~positive regulation of pathway-restricted SMAD protein phosphorylation | | 4 | 2.29E-02 | 2 | 6.93E-02 |
| GO:0030154~cell differentiation | | 11 | 7.07E-02 | 5 | 2.74E-02 |
| GO:0030199~collagen fibril organization | | 4 | 1.31E-02 | 2 | 5.67E-02 |
| GO:0030308~negative regulation of cell growth | | 6 | 1.91E-02 | 3 | 1.49E-02 |
| GO:0030324~lung development | | 13 | 1.94E-10 | 3 | 1.46E-02 |
| GO:0030335~positive regulation of cell migration | | 8 | 9.16E-03 | 3 | 3.66E-02 |
| GO:0030513~positive regulation of BMP signaling pathway | | 6 | 4.07E-05 | 5 | 2.25E-07 |
| GO:0030900~forebrain development | | 4 | 2.05E-02 | 3 | 7.26E-03 |
| GO:0032924~activin receptor signaling pathway | | 4 | 9.98E-04 | 2 | 2.07E-02 |
| GO:0035050~embryonic heart tube development | | 6 | 8.48E-07 | 2 | 3.38E-02 |
| GO:0035116~embryonic hindlimb morphogenesis | | 4 | 5.22E-03 | 3 | 1.10E-03 |
| GO:0035909~aorta morphogenesis | | 5 | 1.65E-05 | 2 | 2.22E-02 |
| GO:0042127~regulation of cell proliferation | | 6 | 8.69E-02 | 5 | 3.38E-04 |
| GO:0042475~odontogenesis of dentin-containing tooth | | 4 | 3.26E-02 | 3 | 3.96E-03 |
| GO:0042493~response to drug | | 8 | 9.23E-02 | 4 | 1.37E-02 |
| GO:0042733~embryonic digit morphogenesis | | 11 | 1.87E-09 | 2 | 9.41E-02 |
| GO:0043066~negative regulation of apoptotic process | | 14 | 5.48E-03 | 4 | 5.12E-02 |
| GO:0043410~positive regulation of MAPK cascade | | 7 | 5.71E-04 | 3 | 1.05E-02 |
| GO:0045669~positive regulation of osteoblast differentiation | | 8 | 1.05E-05 | 5 | 3.01E-06 |
| GO:0045892~negative regulation of transcription, DNA-templated | | 22 | 1.58E-06 | 4 | 5.41E-02 |
| GO:0045893~positive regulation of transcription, DNA-templated | | 38 | 6.23E-18 | 11 | 5.15E-09 |
| GO:0045944~positive regulation of transcription from RNA polymerase II promoter | | 45 | 1.38E-13 | 11 | 8.70E-07 |
| GO:0048844~artery morphogenesis | | 6 | 5.40E-06 | 3 | 6.31E-04 |
| GO:0051145~smooth muscle cell differentiation | | 4 | 5.24E-04 | 2 | 2.66E-02 |
| GO:0055010~ventricular cardiac muscle tissue morphogenesis | | 6 | 1.35E-05 | 2 | 4.10E-02 |
| GO:0060038~cardiac muscle cell proliferation | | 4 | 5.24E-04 | 3 | 2.88E-04 |
| GO:0060045~positive regulation of cardiac muscle cell proliferation | | 6 | 6.92E-06 | 2 | 3.38E-02 |
| GO:0060389~pathway-restricted SMAD protein phosphorylation | | 3 | 1.14E-02 | 2 | 1.92E-02 |
| GO:0060982~coronary artery morphogenesis | | 3 | 3.22E-03 | 2 | 1.34E-02 |
| GO:0072017~distal tubule development | | 3 | 4.76E-04 | 2 | 4.47E-03 |
| GO:0097070~ductus arteriosus closure | | 4 | 1.98E-05 | 4 | 5.92E-08 |
| **GO Cellular Component - Overlap: 9/24 Common, 37.5%** | | **Count** | **p Value** | **Count** | **p Value** |
| GO:0000790~nuclear chromatin | | 9 | 2.17E-03 | 3 | 3.89E-02 |
| GO:0005578~proteinaceous extracellular matrix | | 7 | 9.95E-02 | 3 | 6.95E-02 |
| GO:0005634~nucleus | | 94 | 1.97E-05 | 16 | 6.09E-03 |
| GO:0005654~nucleoplasm | | 71 | 1.97E-10 | 9 | 3.48E-03 |
| GO:0005667~transcription factor complex | | 14 | 5.33E-07 | 3 | 5.16E-02 |
| GO:0005737~cytoplasm | | 78 | 1.48E-02 | 14 | 8.57E-02 |
| GO:0005925~focal adhesion | | 9 | 9.43E-02 | 5 | 1.88E-03 |
| GO:0016020~membrane | | 43 | 1.16E-03 | 15 | 6.11E-02 |
| GO:0043234~protein complex | | 13 | 3.83E-03 | 6 | 1.47E-03 |
| **GO Molecular Function - Overlap: 12/25 Common, 48.0%** | | **Count** | **p Value** | **Count** | **p Value** |
| GO:0000977~RNA polymerase II regulatory region sequence-specific DNA binding | | 11 | 2.63E-04 | 3 | 4.56E-02 |
| GO:0001077~transcriptional activator activity, RNA polymerase II core promoter proximal region sequence-specific binding | | 12 | 1.71E-04 | 4 | 8.15E-03 |
| GO:0001105~RNA polymerase II transcription coactivator activity | | 6 | 8.56E-05 | 3 | 1.82E-03 |
| GO:0003677~DNA binding | | 36 | 1.28E-03 | 7 | 5.92E-02 |
| GO:0003682~chromatin binding | | 27 | 2.57E-12 | 4 | 3.44E-02 |
| GO:0003700~transcription factor activity, sequence-specific DNA binding | | 34 | 8.24E-08 | 5 | 4.53E-02 |
| GO:0003713~transcription coactivator activity | | 9 | 1.18E-02 | 5 | 1.60E-04 |
| GO:0005515~protein binding | | 154 | 1.35E-10 | 16 | 3.20E-04 |
| GO:0008134~transcription factor binding | | 13 | 2.17E-04 | 6 | 1.59E-04 |
| GO:0043565~sequence-specific DNA binding | | 26 | 5.82E-09 | 5 | 1.55E-02 |
| GO:0044212~transcription regulatory region DNA binding | | 18 | 1.21E-09 | 3 | 5.01E-02 |
| GO:0046982~protein heterodimerization activity | | 13 | 1.28E-02 | 4 | 4.41E-02 |
| **KEGG Pathway - Overlap: 10/15 Common, 66.7%** | | **Count** | **p Value** | **Count** | **p Value** |
| hsa04320:Dorso-ventral axis formation | | 4 | 9.48E-03 | 2 | 4.46E-02 |
| hsa04330:Notch signaling pathway | | 6 | 1.07E-03 | 4 | 8.42E-05 |
| hsa04810:Regulation of actin cytoskeleton | | 9 | 2.18E-02 | 3 | 5.58E-02 |
| hsa04919:Thyroid hormone signaling pathway | | 11 | 1.68E-05 | 4 | 1.02E-03 |
| hsa05200:Pathways in cancer | | 14 | 1.19E-02 | 5 | 4.55E-03 |
| hsa05205:Proteoglycans in cancer | | 12 | 4.14E-04 | 4 | 5.32E-03 |
| hsa05206:MicroRNAs in cancer | | 12 | 7.09E-03 | 4 | 1.24E-02 |
| hsa05213:Endometrial cancer | | 4 | 5.34E-02 | 2 | 9.07E-02 |
| hsa05410:Hypertrophic cardiomyopathy (HCM) | | 5 | 3.89E-02 | 3 | 8.75E-03 |
| hsa05412:Arrhythmogenic right ventricular cardiomyopathy (ARVC) | | 4 | 9.73E-02 | 4 | 2.15E-04 |
| **UP_Keywords - Overlap: 11/29 Common, 37.9%** | | **Count** | **p Value** | **Count** | **p Value** |
| Activator | | 33 | 6.73E-13 | 8 | 6.34E-06 |
| Calcium | | 15 | 8.37E-02 | 5 | 1.58E-02 |
| Developmental protein | | 28 | 3.02E-06 | 6 | 5.35E-03 |
| Differentiation | | 14 | 2.58E-02 | 4 | 4.05E-02 |
| EGF-like domain | | 9 | 3.95E-03 | 5 | 1.36E-04 |
| Metal-binding | | 67 | 3.51E-06 | 8 | 9.76E-02 |
| Notch signaling pathway | | 5 | 2.07E-03 | 4 | 2.36E-05 |
| Nucleus | | 93 | 4.70E-08 | 12 | 1.09E-02 |
| Phosphoprotein | | 140 | 1.12E-12 | 15 | 3.82E-02 |
| Transcription | | 66 | 3.43E-13 | 11 | 3.07E-05 |
| Transcription regulation | | 64 | 1.06E-12 | 11 | 2.31E-05 |
| ^1^Number of genes identified within each term | |  |  |  |  |

**Supplementary Table 5: GO, KEGG, and UP Keywords Common Between Human PDA Syndromes and Mouse Models of PDA**

Our list of genetic mouse models of PDA and our OMIM generated list of human single gene syndromes associated with PDA were assessed for predicted biological terms using Gene Ontology Biological Process, Cellular Component, and Molecular Function as well as KEGG Pathway analysis and UniProt Keywords. Resulting terms, counts, and p-values are reported. P-Value in this context refers to the probability that a given number of genes out of the total n genes in a list annotates to a particular GO term, based on the proportion of genes in the genome annotated to that particular GO term.

**Supplemental Methods:**

Transgenic models of PDA in mice were identified by literature search (PubMed, Google Scholar) and examination of mouse phenotype repositories (Mouse Phenome Database, Jackson Laboratories; the International Mouse Phenotyping Consortium) using variations of the following search terms: persistent ductus arteriosus, patent ductus arteriosus, patent arterial duct, or ductus Botalli. Data on human genetic syndromes were extracted from the Online Mendelian Inheritance in Man (OMIM) database (Johns Hopkins University) using similar search terms, resulting in over 450 entries. Filtering the results to those with a phenotypic description and known molecular basis yielded approximately 300 entries. This human gene list was cross-referenced to additional open-access phenotype-based databases (Human Phenotype Ontology and the Monarch Initiative, DisGeNET, FindZebra, GeneReviews, GeneCards/MalaCards, UniProtKB) and manually reviewed to generate a non-redundant list of 224 single-gene syndromes associated with PDA. Mouse and human gene lists were compared for similarity. BioDBnet was used as a resource to identify and convert homologues. A curated human gene list (GeneReviews) was used to avoid overfitting of gene comparisons and provide additional clinical insights. PDA-associated genes in mice and human single-gene syndromes were compared by functional annotation (DAVID, Database for Annotation, Visualization, and Integrated Discovery ^49^).

Candidate effectors identified in human single-gene syndromes were assessed for protein-protein interactions (PPI) through STRING functional proteins association network software v11.0 ^50^. The 224 human genes were input to the STRING database of known and predicted protein-protein interactions, including direct (physical) and indirect (functional) associations. The STRING settings included: 1) full network, 2) all active interaction sources, 3) network edge thickness was set to the confidence score of the PPI, and 4) a minimum interaction score of 0.7, which represents a high confidence level. The resulting network of 219 proteins (nodes) contained 256 interactions (edges) with a PPI enrichment P-value less than 1.0e-16. This network was exported to Cytoscape 3.8.2 for further analysis and visualization. The network was analyzed as an undirected graph and network statistics were generated. Of these, we used betweenness centrality to control node size via continuous mapping. The STRING interaction score was used to control edge thickness, transparency and type (dotted vs solid). In order to visually highlight the strongest associations, solid lines were assigned to interaction scores of 0.9 or higher, and dotted lines to scores less than 0.9. For layout, we used a combination of a forced directed layout on the entire graph as well as yFiles organic edge router. Layout was then manually optimized to minimize label crowding and increase legibility. 71 proteins without high confidence interactions were removed from the graph, resulting in a final network of 148 proteins. The graph was then exported to Adobe Illustrator for final refinements.

**Supplemental Bibliography**

1. Nguyen M, Camenisch T, Snouwaert JN, et al. The prostaglandin receptor EP4 triggers remodelling of the cardiovascular system at birth. *Nature*. Nov 6 1997;390(6655):78-81. <https://doi.org10.1038/36342>.

2. Segi E, Sugimoto Y, Yamasaki A, et al. Patent ductus arteriosus and neonatal death in prostaglandin receptor EP4-deficient mice. *Biochem Biophys Res Commun*. May 8 1998;246(1):7-12. <https://doi.org10.1006/bbrc.1998.8461>.

3. Schneider A, Guan Y, Zhang Y, et al. Generation of a conditional allele of the mouse prostaglandin EP4 receptor. *Genesis*. Sep 2004;40(1):7-14. <https://doi.org10.1002/gene.20048>.

4. Donovan MJ, Hahn R, Tessarollo L, Hempstead BL. Identification of an essential nonneuronal function of neurotrophin 3 in mammalian cardiac development. *Nat Genet*. Oct 1996;14(2):210-3. <https://doi.org10.1038/ng1096-210>.

5. Moser M, Pscherer A, Roth C, et al. Enhanced apoptotic cell death of renal epithelial cells in mice lacking transcription factor AP-2beta. *Genes Dev*. Aug 1 1997;11(15):1938-48. <https://doi.org10.1101/gad.11.15.1938>.

6. Wang J, Ji W, Zhu D, et al. Tfap2b mutation in mice results in patent ductus arteriosus and renal malformation. *J Surg Res*. Jul 2018;227:178-185. <https://doi.org10.1016/j.jss.2018.02.038>.

7. Kirchhoff S, Kim JS, Hagendorff A, et al. Abnormal cardiac conduction and morphogenesis in connexin40 and connexin43 double-deficient mice. *Circ Res*. Sep 1 2000;87(5):399-405. <https://doi.org10.1161/01.res.87.5.399>.

8. Winnier GE, Kume T, Deng K, et al. Roles for the winged helix transcription factors MF1 and MFH1 in cardiovascular development revealed by nonallelic noncomplementation of null alleles. *Dev Biol*. Sep 15 1999;213(2):418-31. <https://doi.org10.1006/dbio.1999.9382>.

9. Kume T, Deng KY, Winfrey V, Gould DB, Walter MA, Hogan BL. The forkhead/winged helix gene Mf1 is disrupted in the pleiotropic mouse mutation congenital hydrocephalus. *Cell*. Jun 12 1998;93(6):985-96. <https://doi.org10.1016/s0092-8674(00)81204-0>.

10. Winnier GE, Hargett L, Hogan BL. The winged helix transcription factor MFH1 is required for proliferation and patterning of paraxial mesoderm in the mouse embryo. *Genes Dev*. Apr 1 1997;11(7):926-40. <https://doi.org10.1101/gad.11.7.926>.

11. Reese J, Paria BC, Brown N, Zhao X, Morrow JD, Dey SK. Coordinated regulation of fetal and maternal prostaglandins directs successful birth and postnatal adaptation in the mouse. *Proc Natl Acad Sci U S A*. Aug 15 2000;97(17):9759-64. <https://doi.org10.1073/pnas.97.17.9759>.

12. Loftin CD, Trivedi DB, Tiano HF, et al. Failure of ductus arteriosus closure and remodeling in neonatal mice deficient in cyclooxygenase-1 and cyclooxygenase-2. *Proc Natl Acad Sci U S A*. Jan 30 2001;98(3):1059-64. <https://doi.org10.1073/pnas.031573498>.

13. Langenbach R, Morham SG, Tiano HF, et al. Prostaglandin synthase 1 gene disruption in mice reduces arachidonic acid-induced inflammation and indomethacin-induced gastric ulceration. *Cell*. Nov 3 1995;83(3):483-92. <https://doi.org10.1016/0092-8674(95)90126-4>.

14. Morham SG, Langenbach R, Loftin CD, et al. Prostaglandin synthase 2 gene disruption causes severe renal pathology in the mouse. *Cell*. Nov 3 1995;83(3):473-82. <https://doi.org10.1016/0092-8674(95)90125-6>.

15. Loftin CD, Trivedi DB, Langenbach R. Cyclooxygenase-1-selective inhibition prolongs gestation in mice without adverse effects on the ductus arteriosus. *J Clin Invest*. Aug 2002;110(4):549-57. <https://doi.org10.1172/JCI14924>.

16. Yu Y, Fan J, Chen XS, et al. Genetic model of selective COX2 inhibition reveals novel heterodimer signaling. *Nat Med*. Jun 2006;12(6):699-704. <https://doi.org10.1038/nm1412>.

17. Coggins KG, Latour A, Nguyen MS, Audoly L, Coffman TM, Koller BH. Metabolism of PGE2 by prostaglandin dehydrogenase is essential for remodeling the ductus arteriosus. *Nat Med*. Feb 2002;8(2):91-2. <https://doi.org10.1038/nm0202-91>.

18. Morano I, Chai GX, Baltas LG, et al. Smooth-muscle contraction without smooth-muscle myosin. *Nat Cell Biol*. Jun 2000;2(6):371-5. <https://doi.org10.1038/35014065>.

19. Kishihara K, Penninger J, Wallace VA, et al. Normal B lymphocyte development but impaired T cell maturation in CD45-exon6 protein tyrosine phosphatase-deficient mice. *Cell*. Jul 16 1993;74(1):143-56. <https://doi.org10.1016/0092-8674(93)90302-7>.

20. Huang J, Cheng L, Li J, et al. Myocardin regulates expression of contractile genes in smooth muscle cells and is required for closure of the ductus arteriosus in mice. *J Clin Invest*. Feb 2008;118(2):515-25. <https://doi.org10.1172/JCI33304>.

21. Long X, Creemers EE, Wang DZ, Olson EN, Miano JM. Myocardin is a bifunctional switch for smooth versus skeletal muscle differentiation. *Proc Natl Acad Sci U S A*. Oct 16 2007;104(42):16570-5. <https://doi.org10.1073/pnas.0708253104>.

22. Ng A, Wong M, Viviano B, et al. Loss of glypican-3 function causes growth factor-dependent defects in cardiac and coronary vascular development. *Dev Biol*. Nov 1 2009;335(1):208-15. <https://doi.org10.1016/j.ydbio.2009.08.029>.

23. Echtler K, Stark K, Lorenz M, et al. Platelets contribute to postnatal occlusion of the ductus arteriosus. *Nat Med*. Jan 2010;16(1):75-82. <https://doi.org10.1038/nm.2060>.

24. Emambokus NR, Frampton J. The glycoprotein IIb molecule is expressed on early murine hematopoietic progenitors and regulates their numbers in sites of hematopoiesis. *Immunity*. Jul 2003;19(1):33-45. <https://doi.org10.1016/s1074-7613(03)00173-0>.

25. Shivdasani RA, Rosenblatt MF, Zucker-Franklin D, et al. Transcription factor NF-E2 is required for platelet formation independent of the actions of thrombopoietin/MGDF in megakaryocyte development. *Cell*. Jun 2 1995;81(5):695-704. <https://doi.org10.1016/0092-8674(95)90531-6>.

26. van der Flier A, Badu-Nkansah K, Whittaker CA, et al. Endothelial alpha5 and alphav integrins cooperate in remodeling of the vasculature during development. *Development*. Jul 2010;137(14):2439-49. <https://doi.org10.1242/dev.049551>.

27. Feng X, Krebs LT, Gridley T. Patent ductus arteriosus in mice with smooth muscle-specific Jag1 deletion. *Development*. Dec 2010;137(24):4191-9. <https://doi.org10.1242/dev.052043>.

28. Holtwick R, Gotthardt M, Skryabin B, et al. Smooth muscle-selective deletion of guanylyl cyclase-A prevents the acute but not chronic effects of ANP on blood pressure. *Proc Natl Acad Sci U S A*. May 14 2002;99(10):7142-7. <https://doi.org10.1073/pnas.102650499>.

29. Kiernan AE, Xu J, Gridley T. The Notch ligand JAG1 is required for sensory progenitor development in the mammalian inner ear. *PLoS Genet*. Jan 2006;2(1):e4. <https://doi.org10.1371/journal.pgen.0020004>.

30. Chang HY, Locker J, Lu R, Schuster VL. Failure of postnatal ductus arteriosus closure in prostaglandin transporter-deficient mice. *Circulation*. Feb 2 2010;121(4):529-36. <https://doi.org10.1161/CIRCULATIONAHA.109.862946>.

31. Zhang M, Chen M, Kim JR, et al. SWI/SNF complexes containing Brahma or Brahma-related gene 1 play distinct roles in smooth muscle development. *Mol Cell Biol*. Jul 2011;31(13):2618-31. <https://doi.org10.1128/MCB.01338-10>.

32. Shen D, Li J, Lepore JJ, et al. Aortic aneurysm generation in mice with targeted deletion of integrin-linked kinase in vascular smooth muscle cells. *Circ Res*. Sep 2 2011;109(6):616-28. <https://doi.org10.1161/CIRCRESAHA.110.239343>.

33. Yajima I, Colombo S, Puig I, et al. A subpopulation of smooth muscle cells, derived from melanocyte-competent precursors, prevents patent ductus arteriosus. *PLoS One*. 2013;8(1):e53183. <https://doi.org10.1371/journal.pone.0053183>.

34. Delmas V, Martinozzi S, Bourgeois Y, Holzenberger M, Larue L. Cre-mediated recombination in the skin melanocyte lineage. *Genesis*. Jun 2003;36(2):73-80. <https://doi.org10.1002/gene.10197>.

35. Tamura M, Hosoya M, Fujita M, et al. Overdosage of Hand2 causes limb and heart defects in the human chromosomal disorder partial trisomy distal 4q. *Hum Mol Genet*. Jun 15 2013;22(12):2471-81. <https://doi.org10.1093/hmg/ddt099>.

36. McGinley AL, Li Y, Deliu Z, Wang QT. Additional sex combs-like family genes are required for normal cardiovascular development. *Genesis*. Jul 2014;52(7):671-86. <https://doi.org10.1002/dvg.22793>.

37. Levet S, Ouarne M, Ciais D, et al. BMP9 and BMP10 are necessary for proper closure of the ductus arteriosus. *Proc Natl Acad Sci U S A*. Jun 23 2015;112(25):E3207-15. <https://doi.org10.1073/pnas.1508386112>.

38. Ricard N, Ciais D, Levet S, et al. BMP9 and BMP10 are critical for postnatal retinal vascular remodeling. *Blood*. Jun 21 2012;119(25):6162-71. <https://doi.org10.1182/blood-2012-01-407593>.

39. Quintero-Rivera F, Xi QJ, Keppler-Noreuil KM, et al. MATR3 disruption in human and mouse associated with bicuspid aortic valve, aortic coarctation and patent ductus arteriosus. *Hum Mol Genet*. Apr 15 2015;24(8):2375-89. <https://doi.org10.1093/hmg/ddv004>.

40. Baeten JT, Jackson AR, McHugh KM, Lilly B. Loss of Notch2 and Notch3 in vascular smooth muscle causes patent ductus arteriosus. *Genesis*. Dec 2015;53(12):738-48. <https://doi.org10.1002/dvg.22904>.

41. Krebs LT, Xue Y, Norton CR, et al. Characterization of Notch3-deficient mice: normal embryonic development and absence of genetic interactions with a Notch1 mutation. *Genesis*. Nov 2003;37(3):139-43. <https://doi.org10.1002/gene.10241>.

42. McCright B, Lozier J, Gridley T. Generation of new Notch2 mutant alleles. *Genesis*. Jan 2006;44(1):29-33. <https://doi.org10.1002/gene.20181>.

43. Krebs LT, Norton CR, Gridley T. Notch signal reception is required in vascular smooth muscle cells for ductus arteriosus closure. *Genesis*. Feb 2016;54(2):86-90. <https://doi.org10.1002/dvg.22916>.

44. Han H, Tanigaki K, Yamamoto N, et al. Inducible gene knockout of transcription factor recombination signal binding protein-J reveals its essential role in T versus B lineage decision. *Int Immunol*. Jun 2002;14(6):637-45. <https://doi.org10.1093/intimm/dxf030>.

45. Tanigaki K, Han H, Yamamoto N, et al. Notch-RBP-J signaling is involved in cell fate determination of marginal zone B cells. *Nat Immunol*. May 2002;3(5):443-50. <https://doi.org10.1038/ni793>.

46. Staiculescu MC, Kim J, Mecham RP, Wagenseil JE. Mechanical behavior and matrisome gene expression in the aneurysm-prone thoracic aorta of newborn lysyl oxidase knockout mice. *Am J Physiol Heart Circ Physiol*. Aug 1 2017;313(2):H446-H456. <https://doi.org10.1152/ajpheart.00712.2016>.

47. Hornstra IK, Birge S, Starcher B, Bailey AJ, Mecham RP, Shapiro SD. Lysyl oxidase is required for vascular and diaphragmatic development in mice. *J Biol Chem*. Apr 18 2003;278(16):14387-93. <https://doi.org10.1074/jbc.M210144200>.

48. Ito S, Yokoyama U, Nakakoji T, et al. Fibulin-1 Integrates Subendothelial Extracellular Matrices and Contributes to Anatomical Closure of the Ductus Arteriosus. *Arterioscler Thromb Vasc Biol*. Sep 2020;40(9):2212-2226. <https://doi.org10.1161/ATVBAHA.120.314729>.

49. Dennis G, Jr., Sherman BT, Hosack DA, et al. DAVID: Database for Annotation, Visualization, and Integrated Discovery. *Genome Biol*. 2003;4(5):P3.

50. Szklarczyk D, Gable AL, Lyon D, et al. STRING v11: protein-protein association networks with increased coverage, supporting functional discovery in genome-wide experimental datasets. *Nucleic Acids Res*. Jan 8 2019;47(D1):D607-D613. <https://doi.org10.1093/nar/gky1131>.
